# Discovery of (*R*)-2-amino-3-triazolpropanoic acid derivatives as NMDA receptor glycine site agonists with GluN2 subunit-specific activity

**DOI:** 10.3389/fchem.2022.1008233

**Published:** 2022-11-17

**Authors:** Fabao Zhao, Georgios Mazis, Feng Yi, James S. Lotti, Michael S. Layeux, Eric P. Schultz, Lennart Bunch, Kasper B. Hansen, Rasmus P. Clausen

**Affiliations:** ^1^ Department of Drug Design and Pharmacology, Faculty of Health and Medical Sciences, University of Copenhagen, Copenhagen, Denmark; ^2^ Department of Medicinal Chemistry, Key Laboratory of Chemical Biology (Ministry of Education), School of Pharmaceutical Sciences, Shandong University, Jinan, Shandong, China; ^3^ Center for Structural and Functional Neuroscience, Center for Biomolecular Structure and Dynamics, Division of Biological Sciences, University of Montana, Missoula, MT, United States

**Keywords:** ionotropic glutamate receptors, ligand-gated ion channel, two-electrode voltage-clamp electrophysiology, co-agonist, subtype selectivity

## Abstract

*N*-Methyl-d-aspartate (NMDA) receptors play critical roles in central nervous system function and are involved in variety of brain disorders. We previously developed a series of (*R*)-3-(5-furanyl)carboxamido-2-aminopropanoic acid glycine site agonists with pronounced variation in activity among NMDA receptor GluN1/2A-D subtypes. Here, a series of (*R*)-2-amino-3-triazolpropanoic acid analogues with a novel chemical scaffold is designed and their pharmacological properties are evaluated at NMDA receptor subtypes. We found that the triazole can function as a bioisostere for amide to produce glycine site agonists with variation in activity among NMDA receptor subtypes. Compounds **13g** and **13i** are full and partial agonists, respectively, at GluN1/2C and GluN1/2D with 3- to 7-fold preference in agonist potency for GluN1/2C-D over GluN1/2A-B subtypes. The agonist binding mode of these triazole analogues and the mechanisms by which the triazole ring can serve as a bioisostere for amide were further explored using molecular dynamics simulations. Thus, the novel (*R*)-2-amino-3-triazolpropanoic acid derivatives reveal insights to agonist binding at the GluN1 subunit of NMDA receptors and provide new opportunities for the design of glycine site agonists.

## 1 Introduction

**FIGURE 1:**
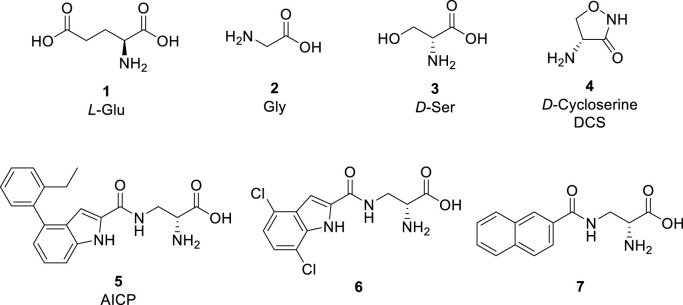
Glu and representative ligands that target the Gly binding site of NMDA receptors.

l-Glutamate (Glu, **1**, Figure 1) is the principle excitatory neurotransmitter in the mammalian brain (Lemoine et al., 2012). When released within the synaptic cleft, Glu binds to both ionotropic and metabotropic Glu receptors, which results in activation and/or modulation of the postsynaptic neuron (Niciu et al., 2012). The ionotropic Glu receptors (iGluRs) are divided into three main functional classes, namely, *N*-methyl-d-aspartate (NMDA) receptors, (*S*)-amino-3-hydroxy-5-methyl-4-isoxazolepropionate (AMPA) receptors, and kainic acid (KA) receptors (Hansen et al., 2021). NMDA receptors play vital roles in learning, memory, and cognitive functions in the normal brain, but dysfunction of NMDA receptors has been associated with a variety of neurological and psychiatric disorders, including pain, stroke, epilepsy, schizophrenia, depression, and various neurodegenerative diseases, such as Parkinson’s and Alzheimer’s diseases (Paoletti et al., 2013; Sanz-Clemente et al., 2013; Yamamoto et al., 2015; Hansen et al., 2021). Therefore, NMDA receptors are desirable therapeutic targets for developing novel treatments in a number of central nervous system disorders.

NMDA receptors are tetrameric ion channels that comprise two GluN1 and two GluN2A−D or two GluN3A-B subunits (Karakas and Furukawa, 2014; Lee et al., 2014). The four GluN2 subunits have distinct developmental and regional expression in the central nervous system and endow NMDA receptor subtypes with different functional properties (Hansen et al., 2018; Hansen et al., 2021). Thus, the physiological roles of NMDA receptor subtypes and their involvement in brain disorders are primarily determined by the GluN2 subunits, and ligands that can discriminate between NMDA receptor subtypes based on the GluN2 subunit are attractive in the development of new therapeutic agents.

The activation of NMDA receptors composed of GluN1 and GluN2 subunits (GluN1/2A-D) requires simultaneous binding of two distinct agonists at the agonist binding domain (ABD), namely Glycine (Gly, **2**, Figure 1) or d-Serine (d-Ser, **3**) to GluN1 and Glu to GluN2 (Benveniste and Mayer, 1991; Clements and Westbrook, 1991; Hansen et al., 2018; Hansen et al., 2021). The endogenous agonists Gly and d-Ser show similar activity at GluN1/2A-D receptors (Figure 1) (Chen et al., 2008; Zhao et al., 2022). By contrast, d-Cycloserine (DCS, **4**) is a full agonist at GluN1/2A (i.e. similar response compared to Gly, R_max_ ≈ 100%), a partial agonist at GluN1/2B and GluN1/2D (i.e., lower response compared to Gly, R_max_ < 100%), and a superagonist at GluN1/2C (i.e., higher response compared to Gly, R_max_ > 100%) (Sheinin et al., 2001; Chen et al., 2008; Dravid et al., 2010; Jessen et al., 2017). Urwyler *et al.* developed amino-3-(4-(2-ethylphenyl)-1*H*-indole-2-carboxamido)propanoic acid (AICP, **5**) as a highly potent NMDA receptor Gly site agonist (Urwyler et al., 2009), that was also identified as a full agonist at GluN1/2A (R_max_ ≈ 100%), a partial agonist at GluN1/2B and GluN1/2D, and a highly efficacious superagonist at GluN1/2C subunit (Jessen et al., 2017; Zhao et al., 2020). Furthermore, we reported a small series of amido-, ether and thioether (*R*)-alanine analogues showing variation among GluN2A-D NMDA receptor subtypes (Maolanon et al., 2017).

**FIGURE 2:**
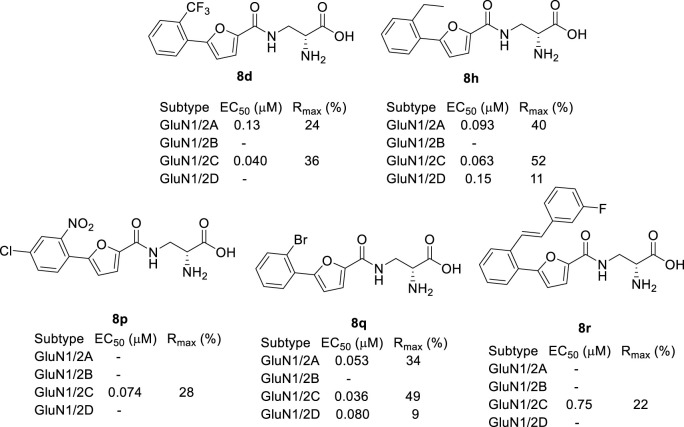
Recently developed NMDA receptor Gly site agonists. EC_50_ is the concentration that produces 50% response at the GluN1 subunit in the continuous presence of a saturating concentration of glutamate. R_max_ is the maximal response compared to the maximal agonist response to Gly. Data are previously published and were measured using two-electrode voltage-clamp recordings of responses from recombinant NMDA receptor subtypes (Zhao et al., 2022).

Based on these studies, we developed a diverse set of Gly site agonists with structural variation in the aromatic part of the amido analogues **5**–**7** (Figure 1). Thus, (R)-3-(5-furanyl)carboxamido-2-aminopropanoic acid derivatives were designed as Gly site agonists that display pronounced variation in potency, efficacy, and GluN2 subunit-specific activity (Figure 2). Among them, **8p** and **8r** are functionally selective GluN1/2C agonists with nanomolar potencies. Besides, compound **8h** and **8q** showed high agonist potencies at GluN1/2A, GluN1/2C and GluN1/2D receptors. Compound **8d** was identified as a partial agonist at GluN1/2A and GluN1/2C receptors (Zhao et al., 2022).

**FIGURE 3:**
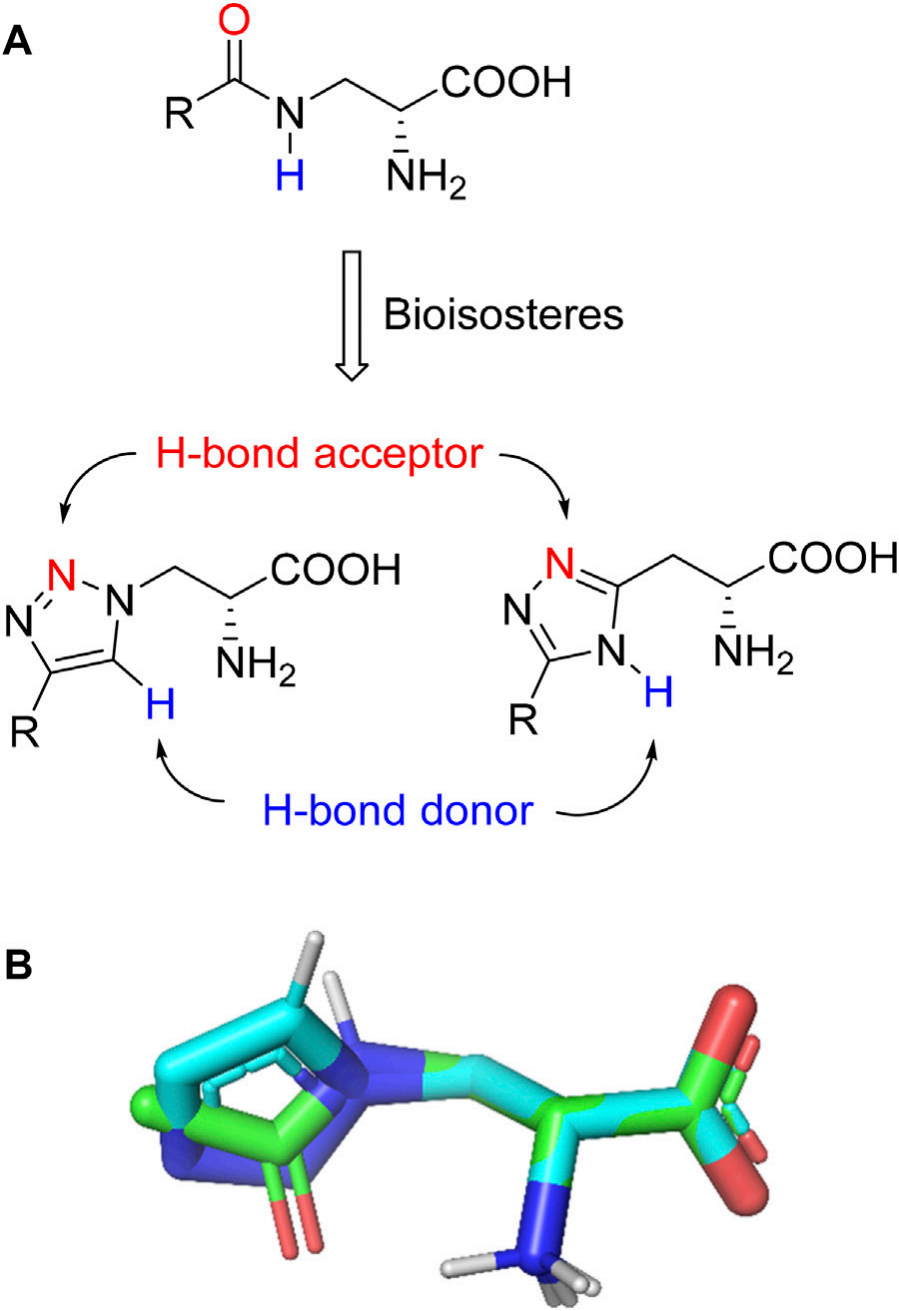
**(A)** Design strategy for novel triazole analogues as NMDA receptor Gly site agonists. **(B)** Overlay of lowest energy conformations for *β*-amide-d-alanine (green) and *β*-triazole-d-alanine (cyan) with amide carbonyl or 1,2,3-triazole. The *C*-4 atom is electronegative, allowing the C-H bond to act as a hydrogen bond donor, and the lone pair of *N*-3 electrons can act as a hydrogen bond acceptor.

Triazoles are well-known chemical scaffolds that have been employed widespread in many biologically active compounds (Sahu et al., 2013). Moreover, the structural features of triazoles enable these chemical scaffolds to mimic different functional groups, justifying the wide use of triazoles as bioisostere of amide bonds in the development of new active molecules (Valverde et al., 2013; Valverde et al., 2015). In this study, we designed a series of 1,4-disubstituted-1,2,3-triazole analogues as NMDA receptor Gly site agonists (Figure 3A). We maintained the essential α-amino acid part of AICP and furan analogues, and replaced the amide bond with the bioisostere 1,2,3-triazole. Different groups were introduced on the *C*-4 position of 1,2,3-triazole ring, including alkyl chains, (hetero)aromatic rings with or without substitutions, and thiophene rings. Despite some differences in the overall dipolar moment and distance between the substituents, their structural features aligned with the amide-binding moiety through overlaying of the lowest energy conformations for *β*-amide-d-alanine and *β*-triazole-d-alanine (Figure 3B). Moreover, the *C*-4 atom is electronegative, allowing the C-H bond to act as a hydrogen bond donor, and the lone pair of *N*-3 electrons can act as a hydrogen bond acceptor. We also replaced the amide bond with 1,2,4-triazole to fulfill the scaffold diversities.

Here, we demonstrate that these new Gly site agonists display variation in activity among GluN1/2A-D NMDA receptor subtypes. These findings expand the pharmacology of GluN1 Gly site agonists and provide a novel scaffold for the development of NMDA receptor ligands.

## 2 Chemistry

**SCHEME 1:**
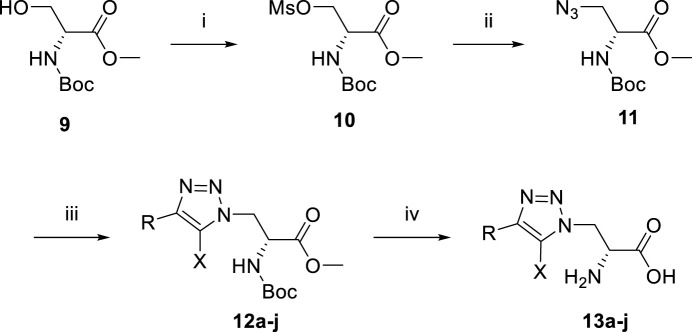
Reagents and conditions: (i) MsCl, NEt_3_, DCM, 0°C. Yield: 61%. (ii) NaN_3_, DMF, 70°C. Yield: 38%. (iii) various alkyne, sodium ascorbate, copper sulfate, *i*-PrOH, H_2_O. (iv) 6M HCl, reflux.

The synthesis of newly designed compounds began with an esterification between *N*-Boc-d-serine methyl ester **9** and methanesulfonyl chloride to give sulfonate **10**, which was then substituted by NaN_3_ to yield the key azide intermediate **11** (Scheme 1). The azide **11** was then treated with various alkynes to afford 1,2,3-triazoles **12a-j**. The Boc and methyl group of **12a**-**j** were removed with 6M HCl and the final pure products **13a**-**j** were obtained by purification using prep-HPLC.

**SCHEME 2:**
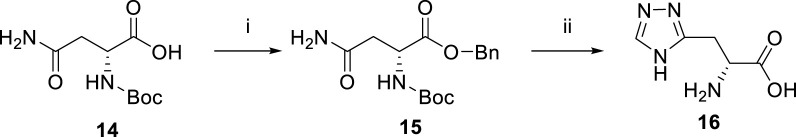
Reagents and conditions: (i) Cs_2_CO_3_, MeOH, rt; then benzyl bromide, DMF, rt. Yield: 60%. (ii) *N*,*N*-dimethylformamide dimethyl acetal, 75°C; then AcOH, hydrazine hydrate; then BBr_3_, DCM, −10°C. Yield: 90%.

The synthesis of compound **16** started from the protection of *N*-Boc-d-asparagine **14** (Scheme 2). The protected intermediate **15** was then treated with *N*,*N*-dimethylformamide dimethyl acetal at 75°C for 2 h. After that, the reaction mixture was redissolved in AcOH and treated with hydrazine hydrate to give the 1,2,4-triazole compound. The protecting groups were cleaved by BBr_3_, and the final product was afforded after purification by prep-HPLC.

For general compounds with a CH-CH_2_ motif, the ^1^H-NMR spectra should give t- and d-peaks for the CH and CH_2_ motif, respectively. Due to the chiral center and the neighboring groups, the α-CH and the β-CH_2_ of the final compounds form two groups of complicated peaks; generally, a broad single peak for the α-proton and multiplets for the β-protons.

## 3 Results and discussion

### 3.1 Pharmacological evaluation

**FIGURE 4:**
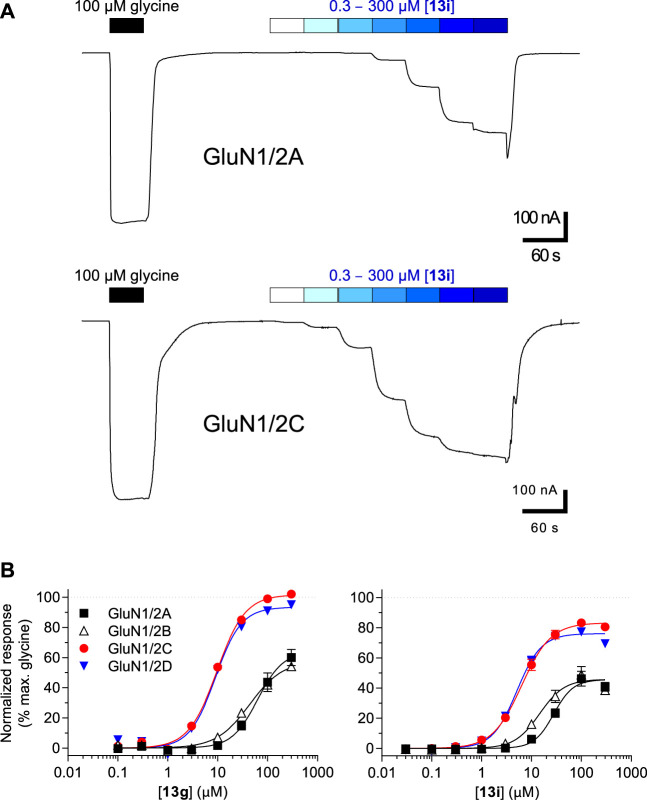
**(A)** Representative recordings of **13i** concentration-response data in the presence of 300 µM Glu at GluN1/2A and GluN1/2C subtypes. Responses were recorded using two-electrode voltage-clamp electrophysiology. **(B)** Concentration-response relationships for **13g** and **13i** at GluN1/2A-D subtypes. The fitted maximal agonist response is normalized to the maximal Gly response. Data from 8–11 oocytes are shown as mean of ± SEM. See Table 1 for EC_50_ values.

The pharmacological properties of analogues **13a**-**j** and **16** were determined with two-electrode voltage-clamp recordings of responses from recombinant NMDA receptor subtypes (Table 1; Figure 4). Compound **13f** was identified as agonist with lower maximal response relative to glycine (R_max_) at GluN1/2A (47%) and GluN1/2B (23%), but R_max_ similar to glycine at GluN1/2C (94%) and GluN1/2D (96%). Similarly, compound **13g** was a partial agonist at GluN1/2A (66%) and GluN1/2B (56%) with a higher agonist efficacy at GluN1/2C (97%) and GluN1/2D (88%). The potency of **13g** was improved compared to **13f**, and **13g** displayed 4- to 7-fold preference in agonist potency for GluN1/2C-D over GluN1/2A-B subtypes. Compound **13i** was a partial agonist at all subtypes with the highest agonist potency in the series and 3- to 6-fold preference in agonist potency for GluN1/2C-D over GluN1/2A-B subtypes. Interestingly, compound **16** was a partial agonist GluN1/2A-C (57%–78%) and a superagonist at GluN1/2D (168%), albeit with low potency compared to Gly (Table 1; Figure 4).

**TABLE 1:** EC_50_ values for activation of rat NMDA receptor subtypes measured using two-electrode voltage-clamp electrophysiology in the presence of 300 µM Glu. Relative efficacy is the fitted maximal response to the agonist compared to maximal response to 100 µM Gly in the same recording. ND means that the EC_50_ could not be determined, but some agonist activity was observed. NR indicates less than 5% response at 100 µM of the agonist. Data are from 3–13 oocytes.^a^ Data for Gly and d-Ser are previously published (Zhao et al., 2022). See Supplementary Table S1 in the Supporting Information for sample sizes, Hill slopes, and standard error of mean.

Compound	R	GluN1/2A		GluN1/2B		GluN1/2C		GluN1/2D	
		EC50 (µM)	Rmax (%)	EC50 (µM)	Rmax (%)	EC50 (µM)	Rmax (%)	EC50 (µM)	Rmax (%)
Gly^a^	—	0.99	100	0.24	100	0.21	100	0.091	100
d-Ser^a^	— 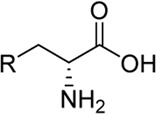	1.0	96	0.62	100	0.19	111	0.15	93
**13a**	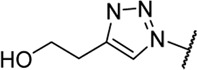	NR		ND		ND		ND	
**13b**	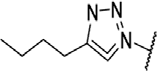	NR		ND		ND		ND	
**13c**	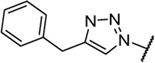	NR		NR		ND		ND	
13d	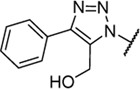	NR		NR		NR		NR	
**13e**	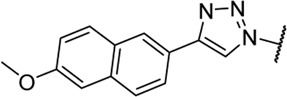	ND		ND		ND		NR	
**13f**	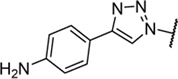	190	47	120	23	32	94	31	96
**13g**	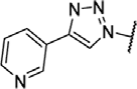	49	66	31	56	8.7	97	7.5	88
**13h**	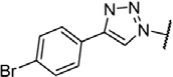	NR		NR		ND		NR	
**13i**	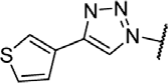	38	56	21	46	6.9	82	5.7	82
**13j**	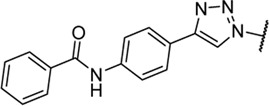	NR		ND		ND		ND	
**16**	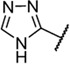	120	57	74	62	94	78	98	168

The structure-activity relationship study shows that pyridine (**13g**) and thiophene rings (**13i**) on the *C*-4 position of the 1,2,3-triazole ring improve potencies. Alkyl chains (**13a** and **13b**), bromobenzene (**13h**), benzyl ring (**13c**) and additional substitutions (**13j**) on the aniline result in inactive compounds or compounds with very low potency.

### 3.2 Molecular modeling

The pharmacological study identified compounds **13g** and **13i** among the new triazole analogues as the most potent Gly site agonists at NMDA receptor subtypes. We therefore performed computational modeling to estimate binding modes for these agonists.

Briefly, a homology model of the GluN1/2C agonist binding domain (ABD) heterodimer was constructed using the GluN1/2A ABD heterodimer crystal structure with bound Gly and Glu (PDB ID: 5I57; Yi et al., 2016) as template. Induced-fit docking of **13g** and **13i** into the Gly binding site in the resulting GluN1/2C model was then performed to obtain docking poses. Subsequently, molecular dynamics simulations (500 ns each) were performed using the best scoring docking pose for each ligand. Finally, the most abundant binding conformations were clustered from the simulation trajectories.

**FIGURE 5:**
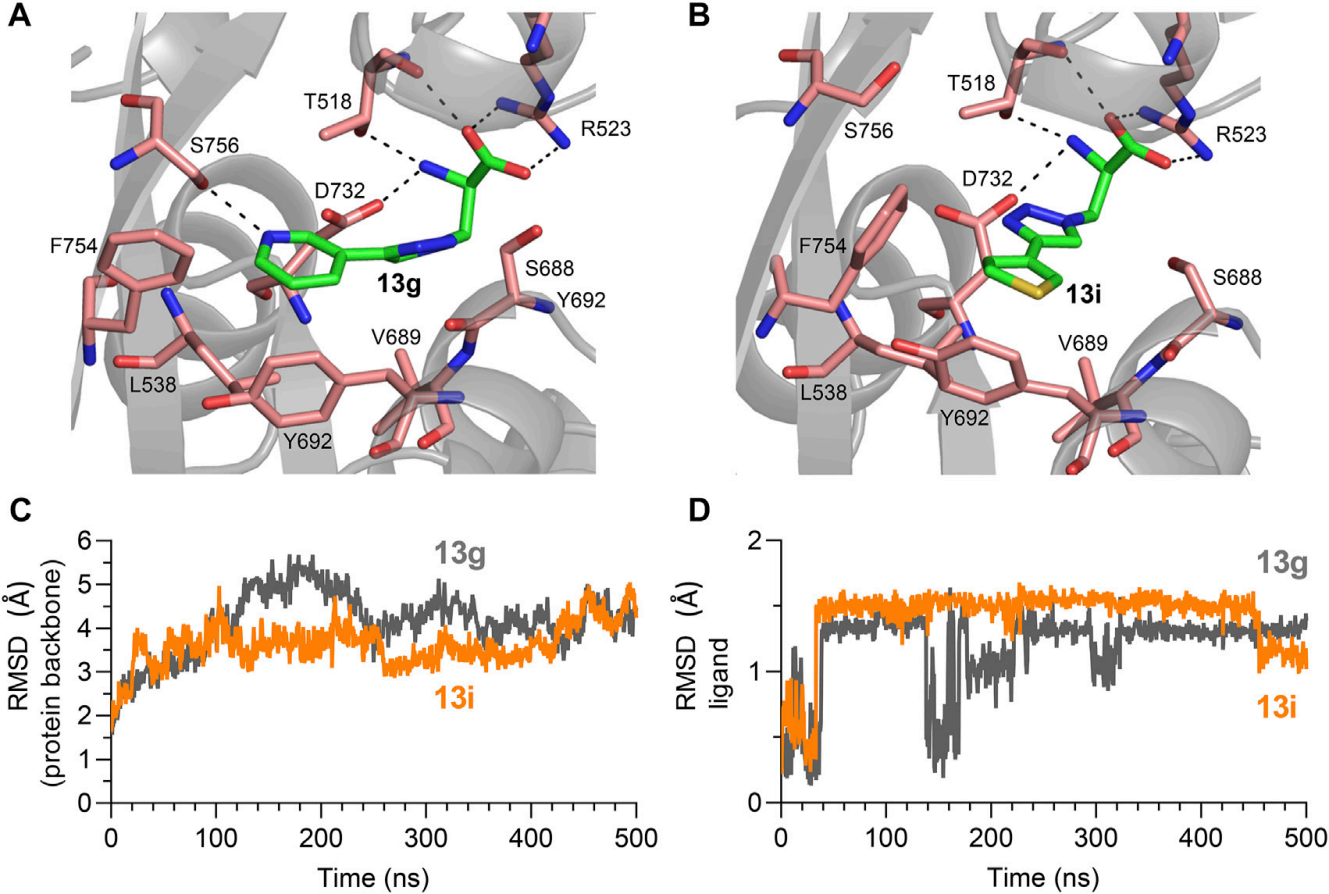
**(A,B)** Most abundant binding mode of **13g** and **13i** in the Gly binding site of GluN1 during molecular dynamics simulation from in initial docking model. **(C,D)** RMSD of protein backbone and ligand compared to the initial docking model for molecular dynamics simulations with **13g** (grey) and **13i** (orange).

In the most abundant conformation for each ligand, the key interactions between receptor and the α-amino acid part were conserved for analogues **13g** and **13i** (Figures 5A,B), and these interactions were identical to interactions observed in GluN1 structures with bound Gly or d-Ser (Furukawa and Gouaux, 2003). GluN1 residues T518, S688 and D732 formed a narrow tunnel in the agonist binding pocket and interacted with the triazole spacers of these analogues via hydrogen bonds, similar to the amide spacer of **8h** and **8p** docked into the GluN1 ABD (Zhao et al., 2022). The pyridine or thiophene ring of analogues **13g** and **13i** interacted with residues Y692 and F754 (GluN1) and bound close to the GluN1 and GluN2 subunits interface (Figures 5A,B). These additional hydrophobic interactions with surrounding residues might improve binding affinity in the series.

Movement of GluN1 L538, V689, Y692, F753, and F754 was required to accommodate binding of the triazole analogues, while the GluN2C ABD remained stable compared to the Gly/Glu-bound GluN1/2C-model structure. Root mean square deviations (RMSD) from the initial docking model remained relatively stable for the protein backbone over the 500 ns simulations (Figure 5C). Similarly, RMSD from the initial docking poses became relatively stable after 30 ns simulation time for both **13g** and **13i** (Figure 5D). The binding conformation of **13i** remained stable for 400 ns, while the binding pose of **13g** was fluctuating according to the RMSD plot, indicating that binding of **13i** is more stable than **13g** at the NMDA receptor Gly binding site. Furthermore, molecular mechanics with generalized born and surface area (MM/GBSA) were calculated to estimate binding free energy for **13g** and **13i** to the Gly binding pocket. The average binding energy is -39.49 kcal/mol and -47.91 kcal/mol for **13g** and **13i**, respectively. These results indicate that binding of **13i** is stronger compared to **13g**, consistent with potencies measured using two-electrode voltage-clamp electrophysiology (Table 1).

## 4 Conclusion

In the present work, a series of triazole analogues (**13a**-**j** and **16**) were designed, synthesized, and characterized at GluN1/2A-D NMDA receptor subtypes. Compounds **13g** and **13i** are full and partial agonists, respectively, at GluN1/2C and GluN1/2D with 3- to 7-fold preference and low micromolar agonist potencies for GluN1/2C-D over GluN1/2A-B subtypes. This profile is different from d-cycloserine (DCS, **4**) in terms of agonist efficacies, but similar in terms of agonist potencies (Sheinin et al., 2001; Chen et al., 2008; Dravid et al., 2010; Jessen et al., 2017). DCS has intriguing neuroactive properties, and administration of DCS can enhance extinction of fear in both rodents and humans, which may be beneficial in the treatment of some psychiatric disorders (Davis et al., 2006; Hofmann et al., 2015; Singewald et al., 2015). However, it remains unresolved whether partial agonism at GluN2B- and GluN2D-containing receptors or superagonism at GluN2C-containing NMDA receptors mediates the behavioral effects of DCS (Sotres-Bayon et al., 2007; Sotres-Bayon et al., 2009; Hillman et al., 2011; Dalton et al., 2012; Gupta et al., 2013; Ogden et al., 2014). To this end, it would desirable to develop novel glycine site NMDA receptor agonists with nanomolar potencies, which would enable effective competition with endogenous glycine or d-serine, but with variation in GluN2 subunit-specific profiles (i.e., agonist efficacies). These novel agonists would enable new studies to determine the optimal GluN2 subunit-specific profiles for the treatment of a range of neurological and psychiatric disorders (Liu et al., 2019; Liu et al., 2021; Shelkar et al., 2021).

The pharmacological results and *in silico* study show that the triazole ring can serve as the bioisostere for the amide in Gly site agonists. Compared to previously reported amido Gly site agonists, such as AICP analogues and furan analogues (Maolanon et al., 2017; Zhao et al., 2022), the new triazole analogues were generally less potent, but more potent than ether and thioether derivatives. However, there are striking similarities between EC_50_ values for **13g** (pyridyl; 7.5–49 µM) and **13i** (thienyl; 5.7–38 µM) and previously published K_i_ values for amido Gly site agonists compounds 8 (pyridyl; 40 µM) and 11 (thienyl; 7.9 µM) in Urwyler *et al.* (Urwyler et al., 2009). Although the methods to evaluate their biological activity are very different, the fact that these compounds (with triazoles or amides) all display activity at micromolar concentrations illustrate that 1,4 triazoles are suitable replacements for the central amide of 2-aminopropionic acid-based NMDA receptor agonists. Thus, triazole derivatives are good starting points for designing new analogues in the development of NMDA receptor glycine site ligands with increased potencies and GluN2 subunit-specific activity.

## 5 Materials and methods

### 5.1 Chemistry

An inert atmosphere of N_2_ using standard Schlenk and syringe-septum technique was applied for reactions requiring inert conditions. All solvents used in reactions were pre-dried with a glass contour solvent system (SG Water, United States LCC). Silica gel 60 (Merck, 0.015–0.040 mm) was used for normal phase column chromatography. Thin layer chromatography (TLC) for monitoring of reactions and chromatography fractions used silica gel 60 F254 aluminum plates (Merck). Reagents and starting materials were purchased from commercial vendors. The ^1^H and ^13^C NMR spectra were generated with Bruker 400 and Bruker 600 spectrometers at 400 and 600 MHz for proton and at 100 and 150 MHz for carbon using signals of residual nondeuterated solvent as internal standards. LC/MS spectra were obtained on an Agilent 1200 system using a Zorbax Eclipse XBD-C^18^ column (4.6 × 50 mm) and a UV detector equipped with an API ion source (HP 1100 MSD). Analytical HPLC spectra were determined on an Ultimate 3000 system with a Gemini-NX C^18^ column (4.6 × 250 mm). Prep-HPLC purification was performed on an Ultimate 3000 system with a Gemini-NX C^18^ column (21 × 250 mm). All compounds were obtained as highly viscous oils, unless otherwise stated. For high-resolution mass spectroscopy (HRMS), compounds were analyzed by ultra-high-performance liquid chromatography (Agilent 1260 UPLC, Agilent Technologies, Santa Clara, CA, United States ) coupled to high-resolution mass spectrometry (6520 Q-TOF, Agilent, United States). Chromatographic separation was carried out using a C^8^ column (Avantor ACE C^8^, 3 μm, 3.0 × 50 mm, Radnor, PA, United States) and a mobile phase constituted of aqueous solution A (water, 0.1% formic acid) and organic solution B (acetonitrile, 0.1% formic acid). The column was held at ambient temperature and a gradient method (1 ml/min) was applied over 6 min, as follows: 0–4 min, 3%–95% B; 4–6 min, 95% B. The autosampler was maintained at ambient temperature and 10 µL of each sample was injected for HRMS analysis in positive mode. Samples were ionized by electrospray with the ionization parameters: capillary voltage, 4.0 kV (positive mode); gas temperature, 325°C; gas flow, 8.0 L/min, fragmentor, 180 V. Data was collected in high-resolution mode (4 GHz) over a mass range of 100–1,700 m/z. Mass correction was employed using reference compounds m/z 322.048121 and 1221.990637.

#### Procedure A: 1,3-Dipolar cycloaddition

3-Azido-*N*-Boc-d-alanine methyl ester **11** (244 mg, 1 mmol) and corresponding alkynes (1 mmol) were dissolved in a mixture of isopropanol and H_2_O (3 ml, v/v, 2:1). Sodium ascorbate (198 mg, 0.1 mmol) was added into the reaction mixture and a solution of CuSO_4_ (8 mg, 0.05 mmol) in H_2_O (0.5 ml) was added later with vigorous stirring. The reaction mixture was then stirred overnight. After concentration, the crude was diluted with H_2_O (15 ml) and extracted with EtOAc (3 × 10 ml). The organic layer was washed by brine twice and the combined organic layers were dried over MgSO_4_ and concentrated in vacuum. The pure product was obtained by dry column vacuum chromatography.

#### Procedure B: Deprotection of the amino acid

The corresponding protected compounds (1 mmol) were dissolved in 6M HCl (5 ml) and stirred overnight under reflux. The aqueous layer was washed with EtOAc (10 ml) and concentrated under vacuum. The crude product was further purified with prep-HPLC to afford the final products as highly viscous oils or amorphous powder without defined melting point.

#### *N*-Boc-*O*-(methylsulfonyl)-d-serine methyl ester (10)

To a solution of *N*-Boc-d-serine methyl ester **9** (2.96 g, 13.5 mmol) in DCM (35 ml) at 0°C, NEt_3_ (3.78 ml, 27 mmol) was added. Then, methanesulfonyl chloride (1.72 ml, 17.6 mmol) was added dropwise. The reaction mixture was stirred for additional 30 min. Saturated NaHCO_3_ (20 ml) was added to quench the reaction and the organic layer was washed with brine 3 times. The combined aqueous layers were extracted with DCM (2 × 25 ml). The combined organic layers were dried over MgSO_4_ and concentrated in vacuum. The crude product was purified by dry column vacuum chromatography to give the title product as yellow oil (2.62 g). ^**1**^ **H NMR** (400 MHz, Chloroform-*d*) δ 5.37 (s, 1H), 4.62–4.59 (m, 1H), 4.58–4.48 (m, 2H), 3.82 (s, 3H), 3.02 (s, 3H), 1.46 (s, 9H). ^**13**^ **C NMR** (101 MHz, Chloroform-*d*) δ 169.2, 155.2, 80.7, 68.9, 60.4, 53.1, 37.5, 28.3. **TLC**: (Hept-EtOAc 6:4) R_f_: 0.31. Yield: 61%.

#### 3-Azido-*N*-Boc-d-alanine methyl ester (11)

To a solution of mesylate serine **10** (100 mg, 0.343 mmol) in anhydrous DMF (5 ml), NaN_3_ (56 mg, 0.858 mmol) was added and heated to 70°C for 10 min. The mixture was transferred to a beaker with cold water (10 ml) and then extracted by EtOAc (3 × 10 ml). The combined organic layers were washed with brine (25 ml), dried over MgSO_4_ and concentrated in vacuum. The crude product was purified by dry column vacuum chromatography to give the title product (32 mg). ^**1**^ **H NMR** (600 MHz, Chloroform-*d*) δ 5.36 (d, *J* = 7.5 Hz, 1H), 4.48 (dt, *J* = 8.1, 3.6 Hz, 1H), 3.80 (s, 3H), 3.72 (d, *J* = 3.8 Hz, 2H), 1.46 (s, 9H). ^**13**^ **C NMR** (151 MHz, Chloroform-*d*) δ 170.4, 155.2, 80.7, 53.7, 53.0, 32.0, 28.4. **TLC**: (Hept-EtOAc 6:4) R_f_: 0.66. Yield: 38%.

#### (*R*)-2-Amino-3-(4-(2-hydroxyethyl)-1*H*-1,2,3-triazol-1-yl)propanoic acid (13a)

Procedure A and B. ^**1**^ **H NMR** (400 MHz, D_2_O) δ 7.86 (s, 1H), 4.98 (dd, *J* = 4.9, 1.9 Hz, 2H), 4.52 (dd, *J* = 5.4, 4.4 Hz, 1H), 3.81 (t, *J* = 6.4 Hz, 2H), 2.90 (t, *J* = 6.3 Hz, 2H). ^**13**^ **C NMR** (101 M Hz, D_2_O) δ 169.2, 125.1, 125.0, 60.4, 53.2, 49.2, 27.5. **LC-MS** m/z [M + H]^+^ calculated for C_7_H_13_N_4_O_3_ 201.10, found 201.3. **HRMS (ESI)** m/z [M + H]^+^ calculated for C_7_H_13_N_4_O_3_ 201.0982, found 201.0987. Yield: 27%, 2 steps.

#### (*R*)-2-Amino-3-(4-butyl-1*H*-1,2,3-triazol-1-yl)propanoic acid (13b)

Procedure A and B. ^**1**^ **H NMR** (600 MHz, D_2_O) δ 8.16 (s, 1H), 5.13 (qd, *J* = 15.3, 4.8 Hz, 2H), 4.72 (t, *J* = 5.2 Hz, 1H), 2.79 (t, *J* = 7.6 Hz, 2H), 1.63 (p, *J* = 7.5 Hz, 2H), 1.30 (h, *J* = 7.4 Hz, 2H), 0.86 (t, *J* = 7.4 Hz, 3H). ^**13**^ **C NMR** (101 MHz, D_2_O) δ 168.6, 125.6, 107.2, 52.6, 49.8, 30.1, 23.3, 21.2, 12.8. **LC-MS** m/z [M + H]^+^ calculated for C_9_H_17_N_4_O_2_ 213.13, found 213.5. **HRMS (ESI)** m/z [M + H]^+^ calculated for C_9_H_17_N_4_O_2_ 213.1346, found 213.1343. Yield: 61%, 2 steps.

#### (*R*)-2-Amino-3-(4-benzyl-1*H*-1,2,3-triazol-1-yl)propanoic acid (13c)

Procedure A and B. ^**1**^ **H NMR** (600 MHz, Methanol-*d*_4_) δ 8.07 (s, 1H), 7.30 (s, 4H), 7.23 (s, 1H), 5.06 (s, 2H), 4.70 (s, 1H), 4.15 (s, 2H). ^**13**^ **C NMR** (151 MHz, Methanol-*d*_4_) δ 168.2, 147.8, 138.6, 129.8, 127.9, 127.4, 53.6, 51.5, 49.4, 49.3, 49.1, 49.0, 48.9, 48.7, 48.6, 31.9. **LC-MS** m/z [M + H]^+^ calculated for C_12_H_15_N_4_O_2_ 247.12, found 247.5. **HRMS (ESI)** m/z [M + H]^+^ calculated for C_12_H_15_N_4_O_2_ 247.1190, found 247.1187. Yield: 37%, 2 steps.

#### (*R*)-2-Amino-3-(5-(hydroxymethyl)-4-phenyl-1*H*-1,2,3-triazol-1-yl)propanoic acid (13d)

Procedure A and B. ^**1**^ **H NMR** (600 MHz, D_2_O) δ 7.56–7.52 (m, 2H), 7.49–7.41 (m, 3H), 5.05 (dd, *J* = 5.4, 3.2 Hz, 2H), 4.78 (s, 2H), 4.76–4.74 (m, 1H). ^**13**^ **C NMR** (151 MHz, D_2_O) δ 176.5, 168.6, 145.6, 133.8, 129.1, 128.6, 127.8, 52.3, 51.0, 47.3. **LC-MS** m/z [M + H]^+^ calculated for C_12_H_15_N_4_O_3_ 263.11, found 263.5. **HRMS (ESI)** m/z C_12_H_15_N_4_O_3_ [M + H]^+^ calculated 263.1139, found 263.1137. Yield: 50%, 2 steps.

#### (*R*)-2-Amino-3-(4-(6-hydroxynaphthalen-2-yl)-1*H*-1,2,3-triazol-1-yl)propanoic acid (13e)

Procedure A and B. ^**1**^ **H NMR** (400 MHz, DMSO-*d*_6_) δ 8.56 (s, 1H), 8.25 (d, *J* = 19.0 Hz, 1H), 7.90–7.80 (m, 2H), 7.76 (dd, *J* = 31.2, 8.5 Hz, 1H), 7.27–7.11 (m, 2H), 5.13 (d, *J* = 4.0 Hz, 2H), 4.77 (s, 1H), 3.31 (s, 3H). ^**13**^ **C NMR** (101 MHz, DMSO-*d*_6_) δ 167.2, 156.0, 147.7, 135.2, 129.5, 128.3, 127.4, 124.4, 124.2, 123.7, 122. 7, 119.2, 108.6, 54.5, 52.4, 49.3. **LC-MS** m/z [M + H]^+^ calculated for C_16_H_17_N_4_O_3_ 313.13, found 313.5. **HRMS (ESI)** m/z [M + H]^+^ calculated for C_16_H_17_N_4_O_3_ 3313.1295, found 313.1294. Yield: 79%, 2 steps.

#### (*R*)-2-Amino-3-(4-(4-aminophenyl)-1*H*-1,2,3-triazol-1-yl)propanoic acid (13f)

Procedure A and B. ^**1**^ **H NMR** (600 MHz, Methanol-*d*_4_) δ 8.55 (s, 1H), 8.04 (d, *J* = 8.5 Hz, 2H), 7.52 (d, *J* = 8.5 Hz, 2H), 5.10 (d, *J* = 5.0 Hz, 2H), 4.74 (t, *J* = 5.0 Hz, 1H). ^**13**^ **C NMR** (151 MHz, Methanol-*d*_4_) δ 168.6, 147.6, 132.7, 131.8, 128.4, 124.9, 124.4, 53.7, 50.2. **LC-MS** m/z [M + H]^+^ calculated for C_11_H_14_N_5_O_2_ 248.11, found 248.4. **HRMS (ESI)** m/z [M + H]^+^ calculated for C_11_H_14_N_5_O_2_ 248.1142, found 248.1142. Yield: 72%, 2 steps.

#### (*R*)-2-Amino-3-(4-(pyridin-3-yl)-1*H*-1,2,3-triazol-1-yl)propanoic acid (13g)

Procedure A and B. ^**1**^ **H NMR** (600 MHz, D_2_O) δ 9.27 (d, *J* = 2.0 Hz, 1H), 9.01 (dt, *J* = 8.3, 1.7 Hz, 1H), 8.82 (dt, *J* = 5.8, 1.1 Hz, 1H), 8.69 (d, *J* = 2.0 Hz, 1H), 8.21 (dd, *J* = 8.3, 5.8 Hz, 1H), 5.23–5.14 (m, 2H), 4.65–4.60 (m, 1H). ^**13**^ **C NMR** (151 MHz, D_2_O) δ 169.4, 143.3, 141.7, 140.4, 138. 2, 130.1, 127.8, 125.4, 49.6. **LC-MS** m/z [M + H]^+^ calculated for C_10_H_12_N_5_O_2_ 234.10, found 234.5. **HRMS (ESI)** m/z [M + H]^+^ calculated for C_10_H_12_N_5_O_2_ 234.0986, found 234.0986. Yield: 65%, 2 steps.

#### (*R*)-2-Amino-3-(4-(4-bromophenyl)-1*H*-1,2,3-triazol-1-yl)propanoic acid (13h)

Procedure A and B. ^**1**^ **H NMR** (600 MHz, Methanol-*d*_4_) δ 8.39 (s, 1H), 7.77 (d, *J* = 8.5 Hz, 2H), 7.62 (d, *J* = 8.5 Hz, 2H), 5.09–5.03 (m, 2H), 4.67 (t, *J* = 4.7  Hz, 1H). ^**13**^ **C NMR** (151 MHz, Methanol-*d*_4_) δ 168.7, 148.3, 133.2, 130.6, 128.5, 123.7, 123.4, 53.8, 50.2, 49.4, 49.3, 49.1, 49.0, 48.9, 48.7, 48.6. **LC-MS** m/z [M + H]^+^ calculated for C_11_H_12_BrN_4_O_2_ 311.01, found 311.4. **HRMS (ESI)** m/z [M + H]^+^ calculated for C_11_H_12_BrN_4_O_2_ 311.0138, found 311.0133. Yield: 94%, 2 steps.

#### (*R*)-2-Amino-3-(4-(thiophen-3-yl)-1*H*-1,2,3-triazol-1-yl)propanoic acid (13i)

Procedure A and B. ^**1**^ **H NMR** (600 MHz, Methanol-*d*_4_) δ 8.29 (s, 1H), 7.81–7.77 (m, 1H), 7.56–7.48 (m, 2H), 5.08–5.03 (m, 2H), 4.71 (t, *J* = 5.0 Hz, 1H). ^**13**^ **C NMR** (151 MHz, Methanol-*d*_4_) δ 168.6, 145.6, 132.5, 127.8, 126.7, 123.3, 122.6, 53.8, 50.1, 49.4, 49.3, 49.1, 49.0, 48.9, 48.7, 48.6. **LC-MS** m/z [M + H]^+^ calculated for C_9_H_11_N_4_O_2_S 239.06, found 239.5. **HRMS (ESI)** m/z [M + H]^+^ calculated for C_9_H_11_N_4_O_2_S 239.0597, found 239.0592. Yield: 80%, 2 steps.

#### (*R*)-2-Amino-3-(4-(4-benzamidophenyl)-1*H*-1,2,3-triazol-1-yl)propanoic acid (13j)

Procedure A and B. ^**1**^ **H NMR** (600 MHz, Methanol-*d*_4_) δ 8.37 (s, 1H), 7.94 (d, *J* = 7.3 Hz, 2H), 7.83 (q, *J* = 8.3, 7.8 Hz, 4H), 7.58 (t, *J* = 7.2 Hz, 1H), 7.51 (t, *J* = 7.3 Hz, 2H), 5.07 (s, 2H), 4.71 (s, 1H). ^**13**^ **C NMR** (151 MHz, Methanol-*d*_4_) δ 168.9, 168.6, 148.9, 140.3, 136.1, 132.9, 129.6, 128.6, 127.3, 127.3, 123.4, 122.4, 53.9, 50.4, 49.4, 49.3, 49.1, 49.0, 48.9, 48.7, 48.6. **LC-MS** m/z [M + NH_4_]^+^ calculated C_18_H_21_N_6_O_3_ 369.17, found 369.6. **HRMS (ESI)** m/z [M + H]^+^ calculated for C_18_H_18_N_5_O_3_ 352.1404, found 352.1407. Yield: 72%, 2 steps.

#### Benzyl-*N*-Boc-d-asparaginate (15)

*N*-Boc-d-asparagine (1 g, 1 eq) was dissolved in MeOH (20 ml) and aqueous Cs_2_CO_3_ solution (10 ml, 20% w/v) was added. After stirring for 1 h, the solvent was removed and re-dissolved in DMF (20 ml). Benzyl bromide (770 μL, 1.5 eq) was added into the mixture and stirred overnight at rt. The solution was concentrated, and diluted in water (50 ml) to form a precipitate. The precipitate was then collected, dissolved in EtOAc (20 ml) and washed with water (10 ml) to afford the final product **16** as a white powder (830 mg, 60%). ^**1**^ **H NMR** (600 MHz, Methanol-*d*_4_) δ 7.37–7.29 (m, 5H), 5.16 (s, 2H), 4.51 (d, J = 6.2 Hz, 1H), 2.73 (d, *J* = 5.9 Hz, 2H), 1.42 (s, 9H). ^**13**^ **C NMR** (151 MHz, Methanol-*d*_4_) δ 174.9, 173.1, 157.8, 137.2, 129.5, 129.2, 129.1, 80.8, 68.1, 51.9, 49.6, 37.9, 28.7. **LC-MS** m/z [M + H-Boc]^+^ calculated for C_11_H_15_N_2_O_3_ 223.11, found 223.5. Yield: 60%.

#### (*R*)-2-Amino-3-(4*H*-1,2,4-triazol-3-yl)propanoic acid (16)

A solution of benzyl-*N*-Boc-d-asparaginate **16** (100 mg, 1 eq) and DMF-DMA (4 ml) was stirred at 75°C for 2 h. The mixture was concentrated and redissolved in AcOH (5 ml). To the resulting solution, hydrazine hydrate (35.3 μL, 2 eq) was added and stirred overnight at rt. The solution was then concentrated to dryness. The crude was dissolved in anhydrous DCM (10 ml), and cooled to −10°C under N_2_ atmosphere. BBr_3_ (5 ml) was then added dropwise and stirred for another 2 h. The resulting mixture was dissolved in methanol (10 ml) and further purified by prep-HPLC. ^**1**^ **H NMR** (600 MHz, Methanol-*d*_4_) δ 8.85 (s, 1H), 4.49 (dd, *J* = 7.4, 5.3 Hz, 1H), 3.55–3.42 (m, 2H). ^**13**^ **C NMR** (151 MHz, Methanol-*d*_4_) δ 170.2, 156.6, 145.6, 52.2, 49.6, 28.4. **LC-MS** m/z [M + H]^+^ calculated for C_5_H_9_N_4_O_2_ 157.07, found 157.4. **HRMS (ESI)** m/z [M + H]^+^ calculated for C_5_H_9_N_4_O_2_ 157.0720, found 157.0719. Yield: 90%.

### 5.2 Pharmacological evaluation

For expression in *Xenopus laevis* oocytes purchased from Rob Weymouth (*Xenopus* 1, Dexter, MI), cDNAs encoding rat NMDA receptor subunits, GluN1-1a (Genbank accession number U11418 and U08261), GluN2A (D13211), GluN2B (U11419), GluN2C (M91563), and GluN2D (L31611), were used to produce cRNAs using the mMessage mMachine kit (Ambion, Life Technologies, Paisley, United Kingdom) according to the manufacturer’s protocol. The open reading frame of the GluN2B subunit was altered without changing the amino acid sequence to remove a termination site for the T7 RNA polymerase used to synthesize cRNA (Hansen et al., 2014).

Oocytes were injected with cRNAs for GluN1 and GluN2 subunits at a 1:2 ratio, and incubated as previously described (Hansen et al., 2013). The injected oocytes were used for two-electrode voltage-clamp recordings at room temperature (21–23°C) after 2–4 days of expression. as previously described (Hansen et al., 2013). The holding potential was −40 mV in all recordings. The recording solution was comprised of (in mM) 90 NaCl, 1 KCl, 10 HEPES, 0.5 BaCl_2_ and 0.01 EDTA (pH 7.4). For experiments with GluN1/2A or GluN1/2B receptors, the oocytes were injected at least 10 min before recordings with 50 mM BAPTA (30–50 nL) to minimize use-dependent increases in response amplitudes (Williams, 1993). Compounds were dissolved as 20–100 mM stock solutions in DMSO, and DMSO concentrations were constant in all recording solutions and never exceeded 1%.

GraphPad Prism (GraphPad Software, La Jolla, CA) was used to analyze agonist concentration-response data. Briefly, the data for each oocyte were fitted to the following Hill equation, I = R_max_/(1 + 10^((logEC_50_-log [A])*nH)). R_max_ is the maximum agonist current response normalized to the maximal response to 100 µM Gly, nH is the Hillslope, [A] is the concentration of agonist, and EC_50_ is the concentration of agonist that activates a half-maximum response.

### 5.3 Molecular modeling

Computational modeling experiments were performed in the Schrödinger suite, Release 2019–1, Maestro version 11.9.011.

The full-length model of GluN2C (AlphaFold, AF-Q14957-F1) was downloaded from Uniport (ID: Q14957), which was then aligned with the GluN1/2A ABD crystal structure (PDB ID: 5I57) with PyMol. The GluN2C ABD domain (amino acids 401–535, 660–798) was extracted, and combined with GluN1 and ligands (Gly and Glu). This GluN1/2C ABD heterodimer model was then prepared using the Protein Preparation Wizard with default parameters and the resulting protein structure was used for induced-fit docking.

Compounds were prepared using the LigPrep module with default parameters. The highest scoring conformation from the LigPrep results was used for conformational search, which was performed with OPLS3e force field and H_2_O as solvent. Charges were obtained from force field and cutoff was set to extended. No constrains and substructures was used and the Mini method was set to PRCG (Polak-Ribier Conjugate Gradient; Polak and Ribiere, 1969) with 2500 maximum interactions. Convergence was set to Gradient with the threshold as 0.05. The conformational search method was set to mixed torsional/low-mode sampling. And the search was customized with intermediate torsion sampling option. Maximum number of steps were set to 1000 and 100 steps were set per rotatable bond. All other options and parameters were set as default.

Induced-fit docking of compound **13g** and **13i** were performed with default parameters. XP Glide Redocking Precision was applied as the Glide redocking precision and the other parameters was set as default. All docking results were treated with energy minimization, and the binding pose of each compound was selected based on alignment with Gly and the IFDScore.

Molecular dynamic simulations were performed with the Desmond module of Schrödinger Suites. The highest scoring docking pose of each compound was used as the initial input structure, and the docking complex was solvated into a TIP3P orthorhombic water box. The distance between box edge and the protein was set to ≥10 Å. The whole system was neutralized with corresponding number of Na^+^ or Cl^−^, and 150 mM NaCl was added into the system. The production simulation was performed with NPT (constant particle number, pressure and temperature) ensemble at 300 K and 1.0 bar. The whole system was relaxed before production simulation and all other parameters were set as default. The most abundant binding conformations were generated by clustering the production trajectory. The RMSD plots of ligands and protein backbone were generated via the simulation event analysis panel of Maestro with all hydrogens neglected, and the MM/GBSA of the protein-ligand complexes were calculated.

Amino acid residues are numbered based on the full-length polypeptide sequence, including the signal peptide (initiating methionine is 1).

## Data Availability

The original contributions presented in the study are included in the article/Supplementary Material, further inquiries can be directed to the corresponding authors.
