# Supplementary figures and images for: Discovery of (R)-2-amino-3-triazolpropanoic acid derivatives as NMDA receptor glycine site agonists with GluN2 subunit-specific activity
Source: Front Chem. 2022 Nov 17;10:1008233. doi: 10.3389/fchem.2022.1008233 (PMC9713482; doi:10.3389/fchem.2022.1008233)

10


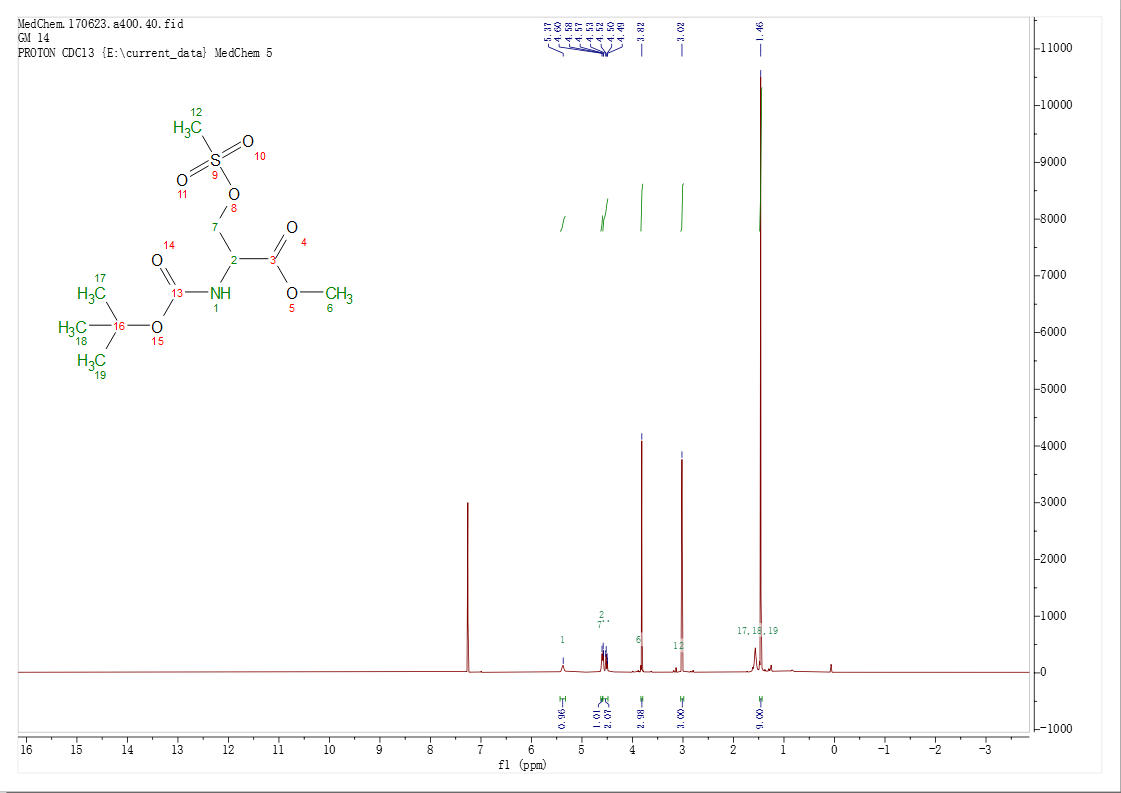


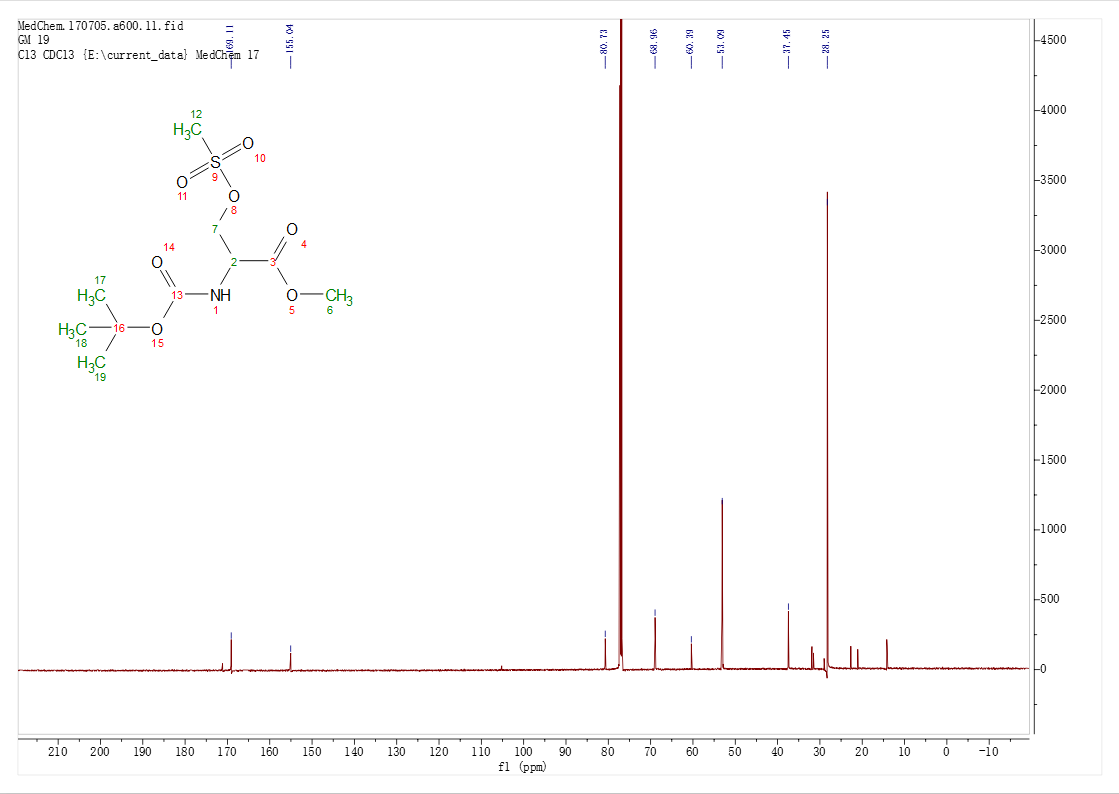


11


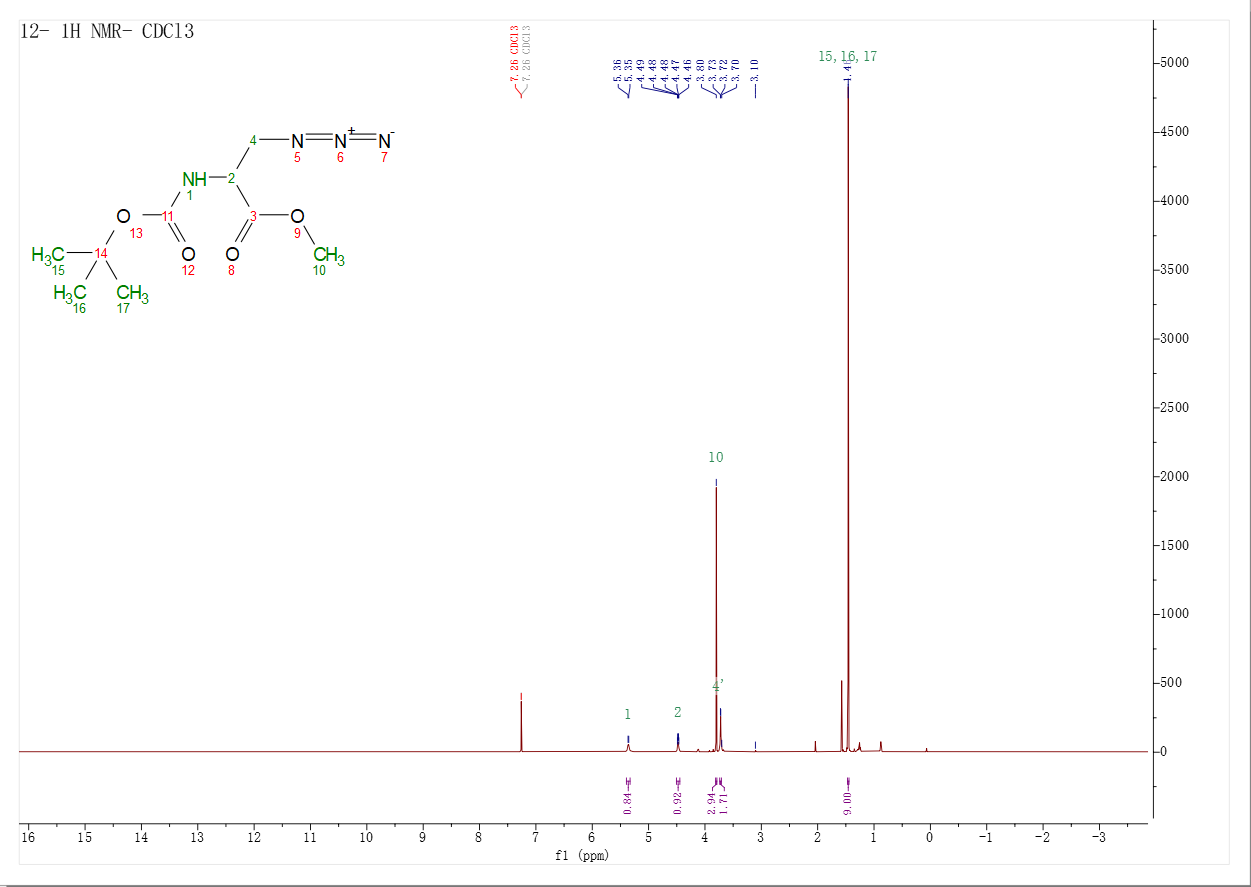


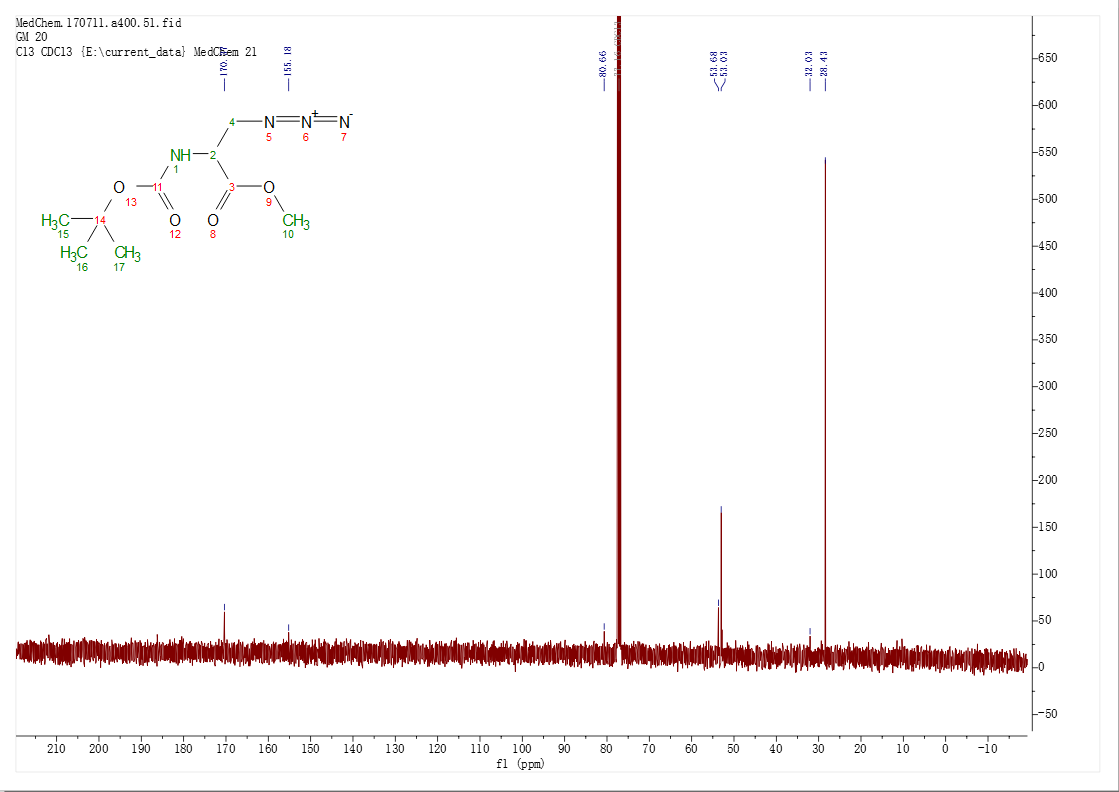


13a


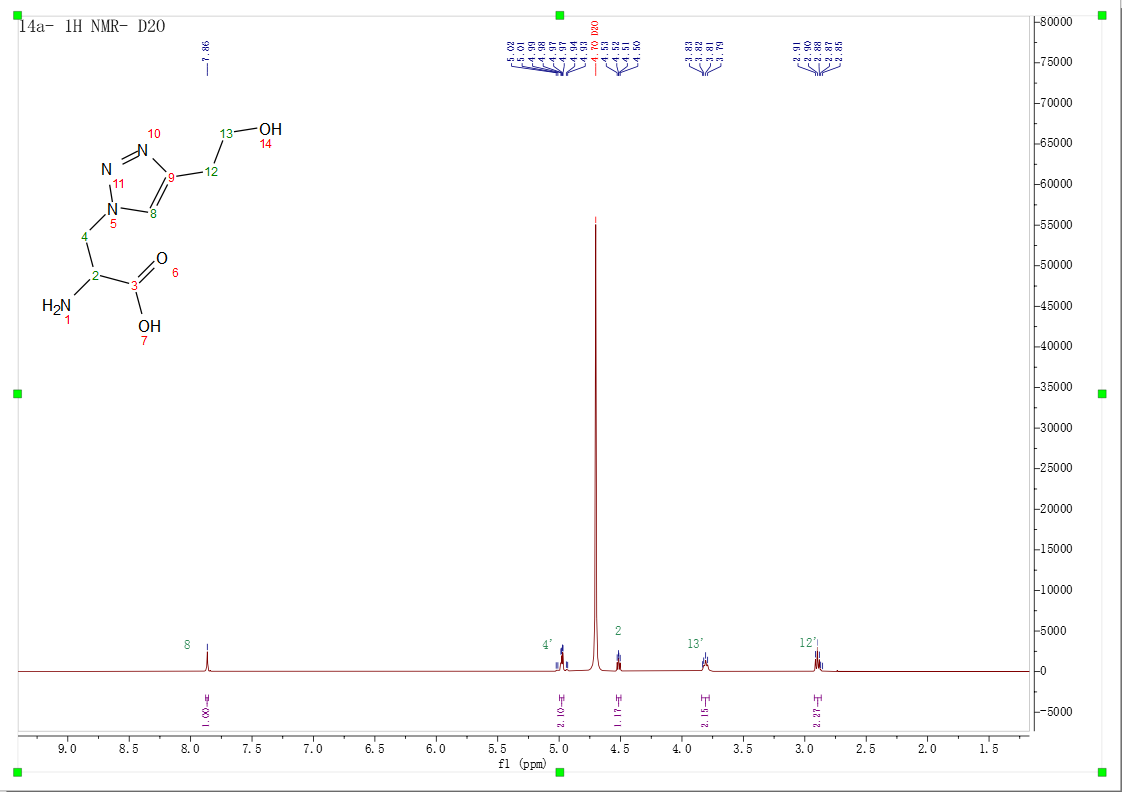


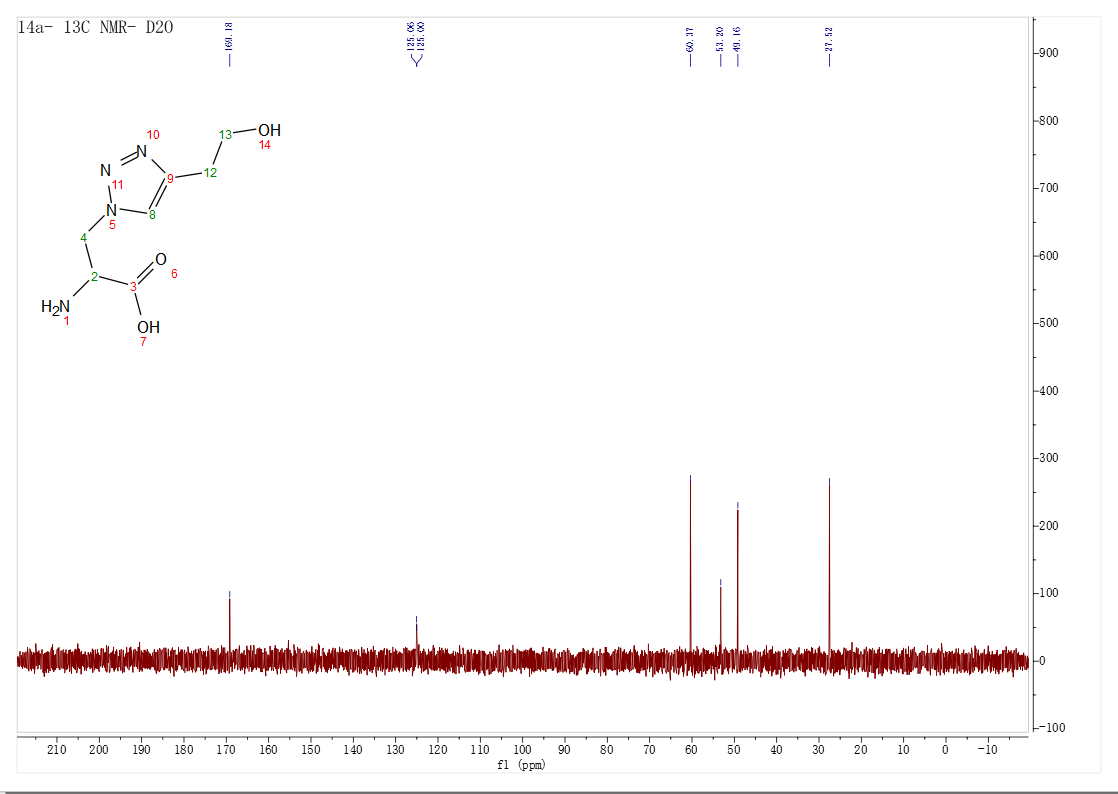


13b


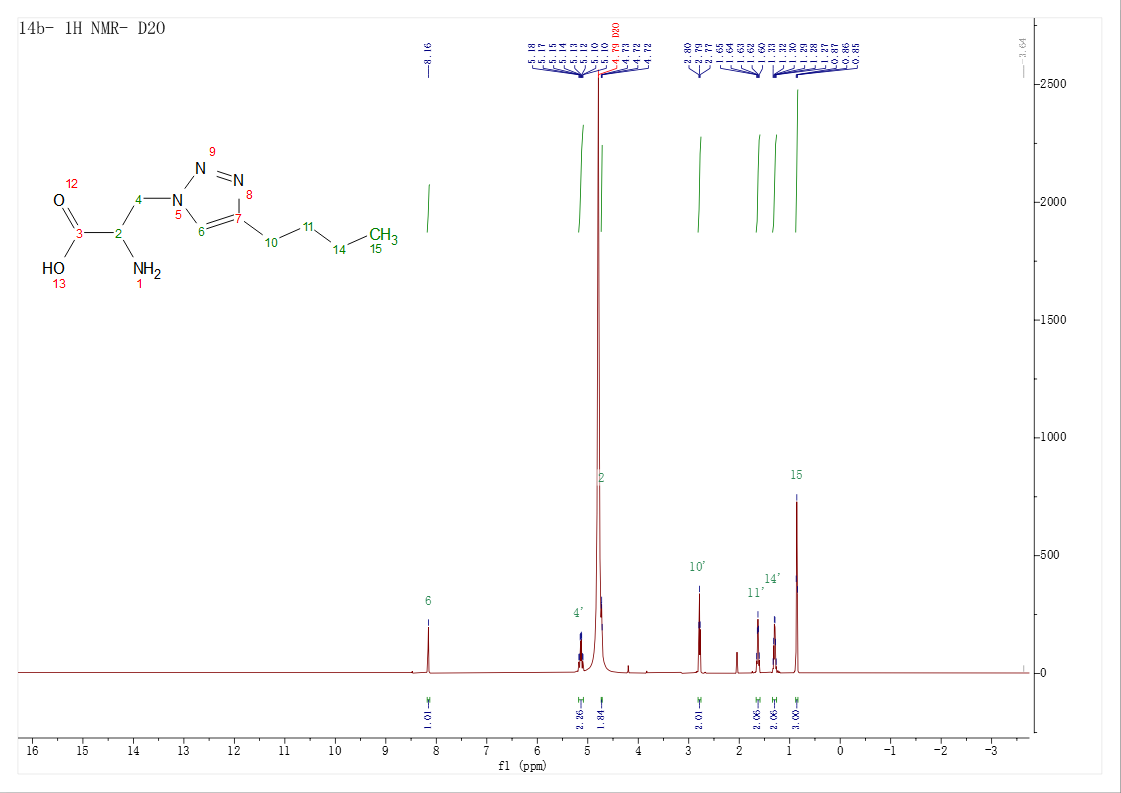


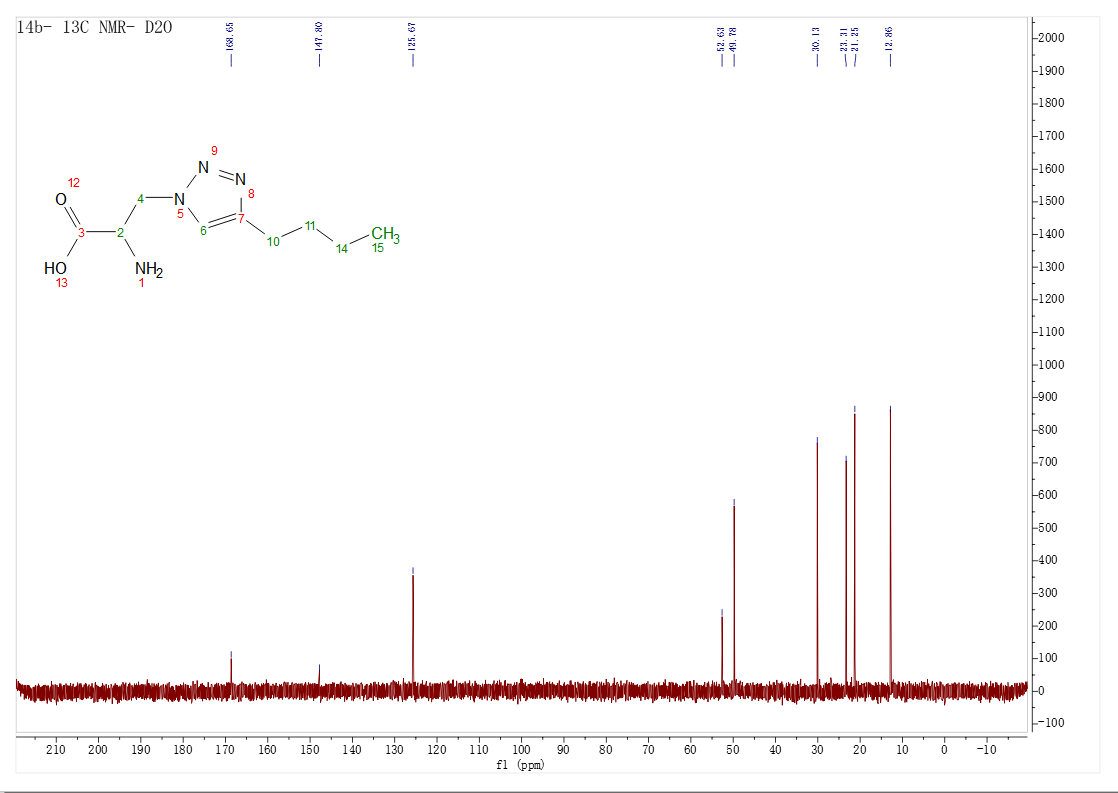


13c


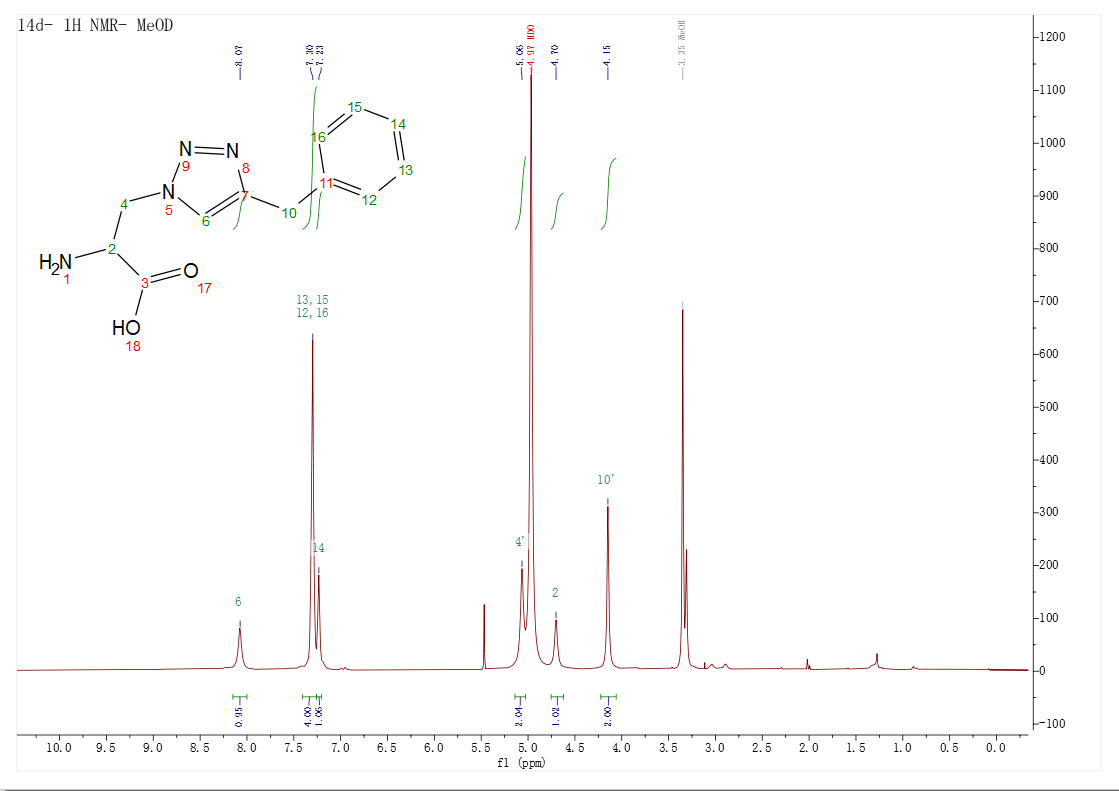


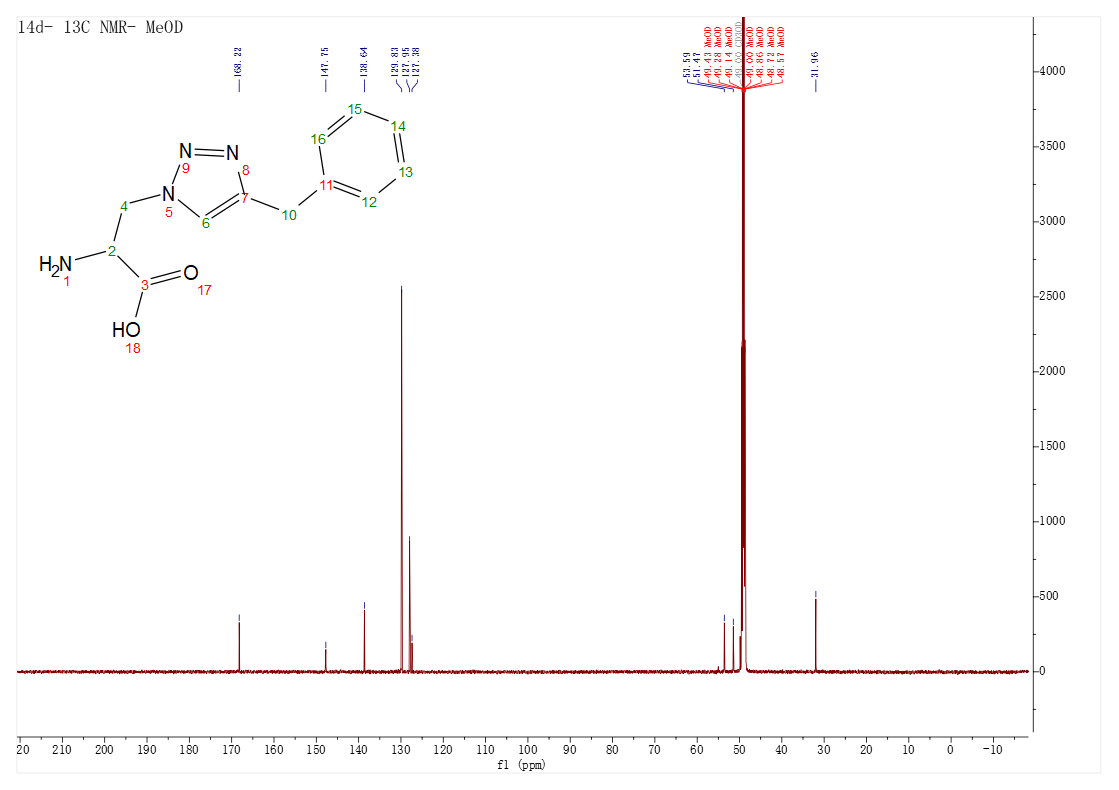


13d


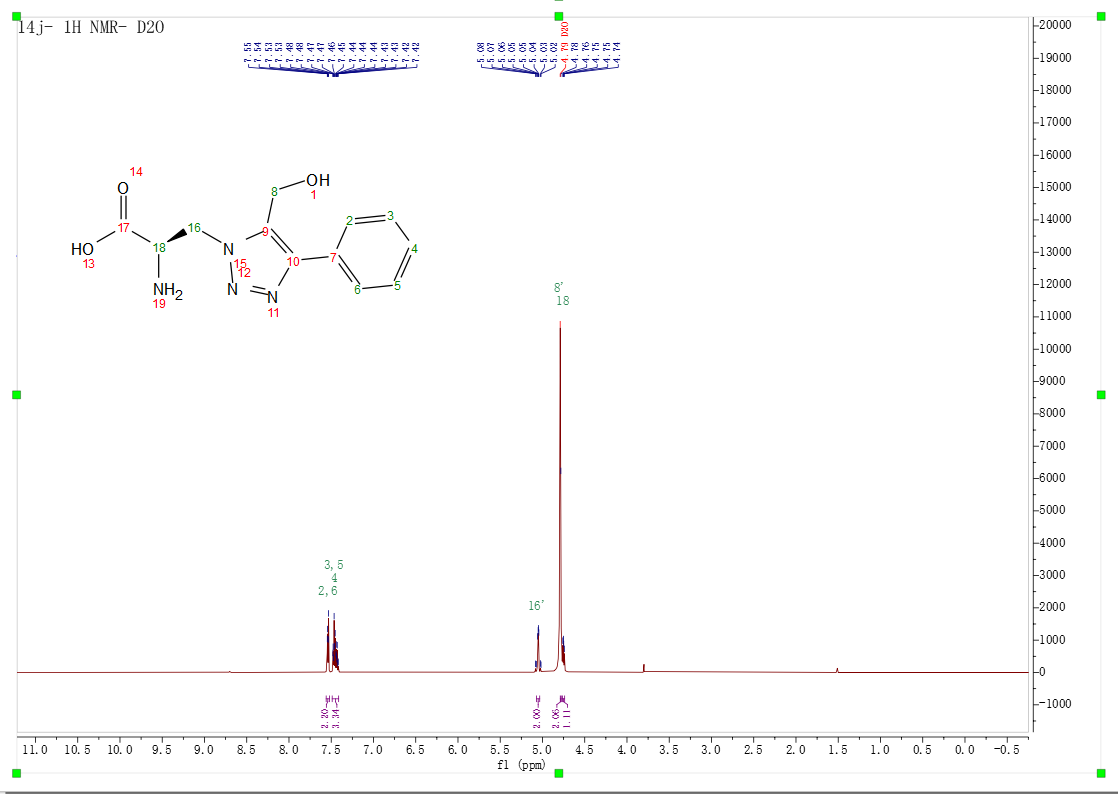


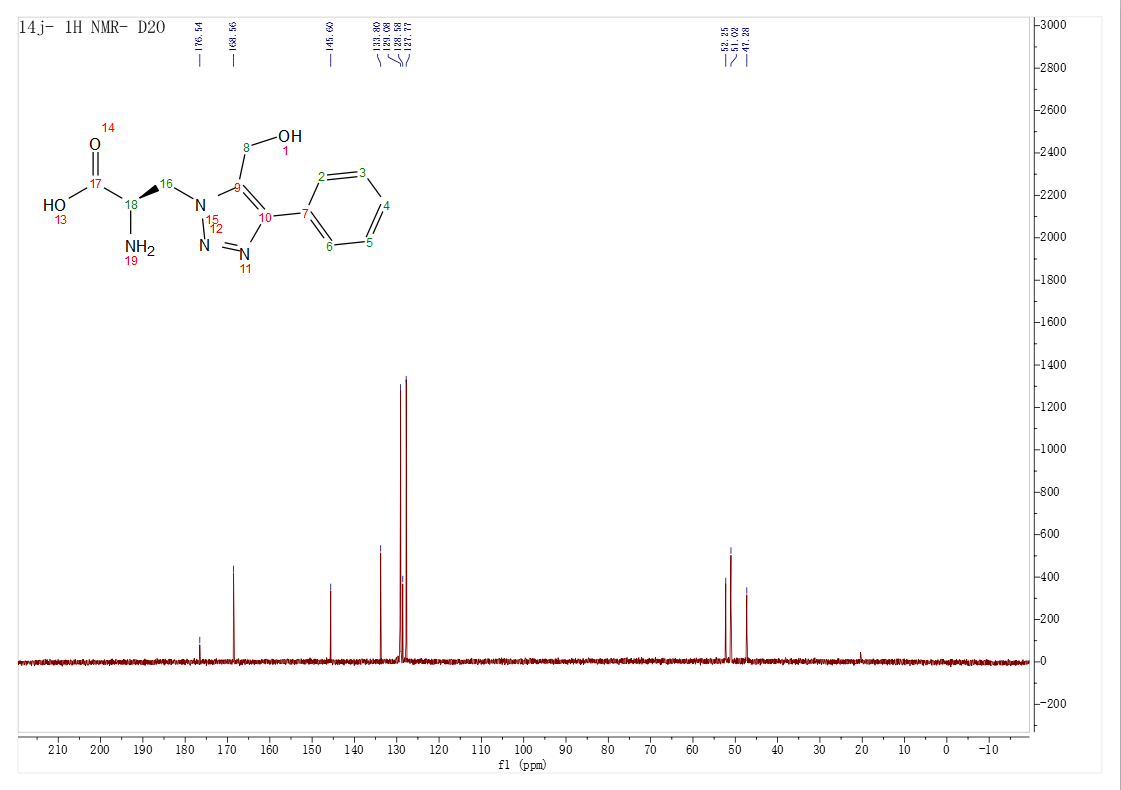


13e


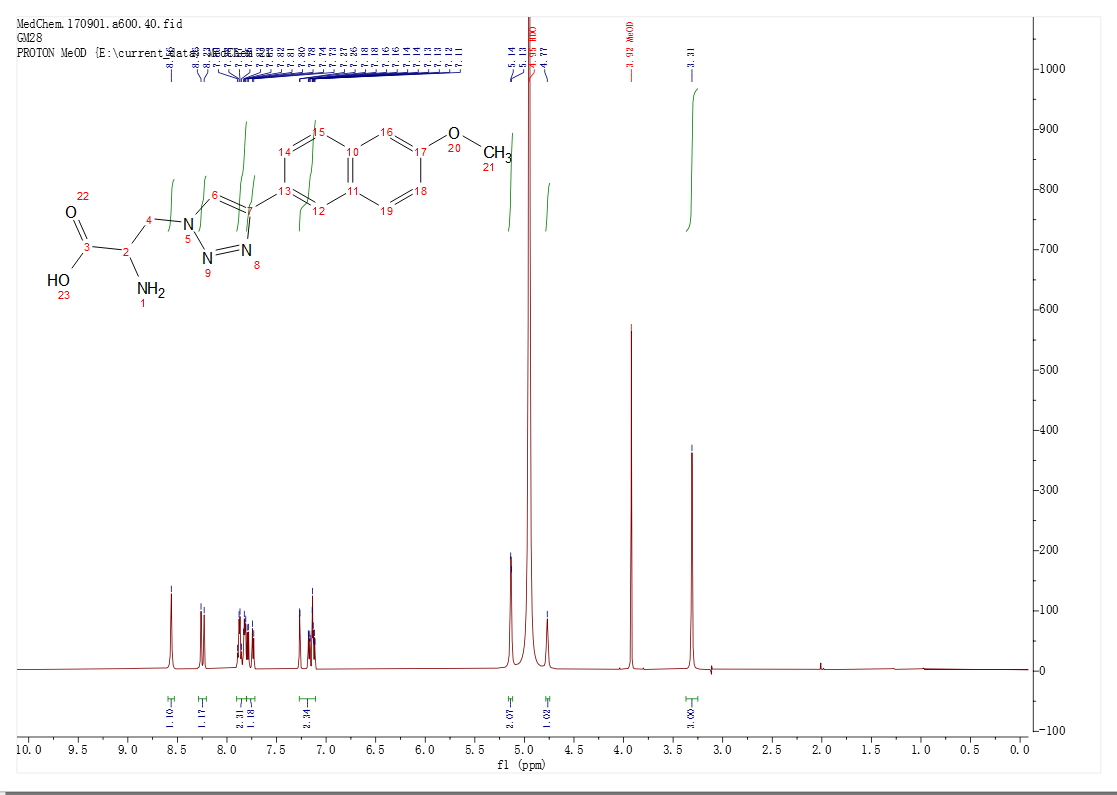


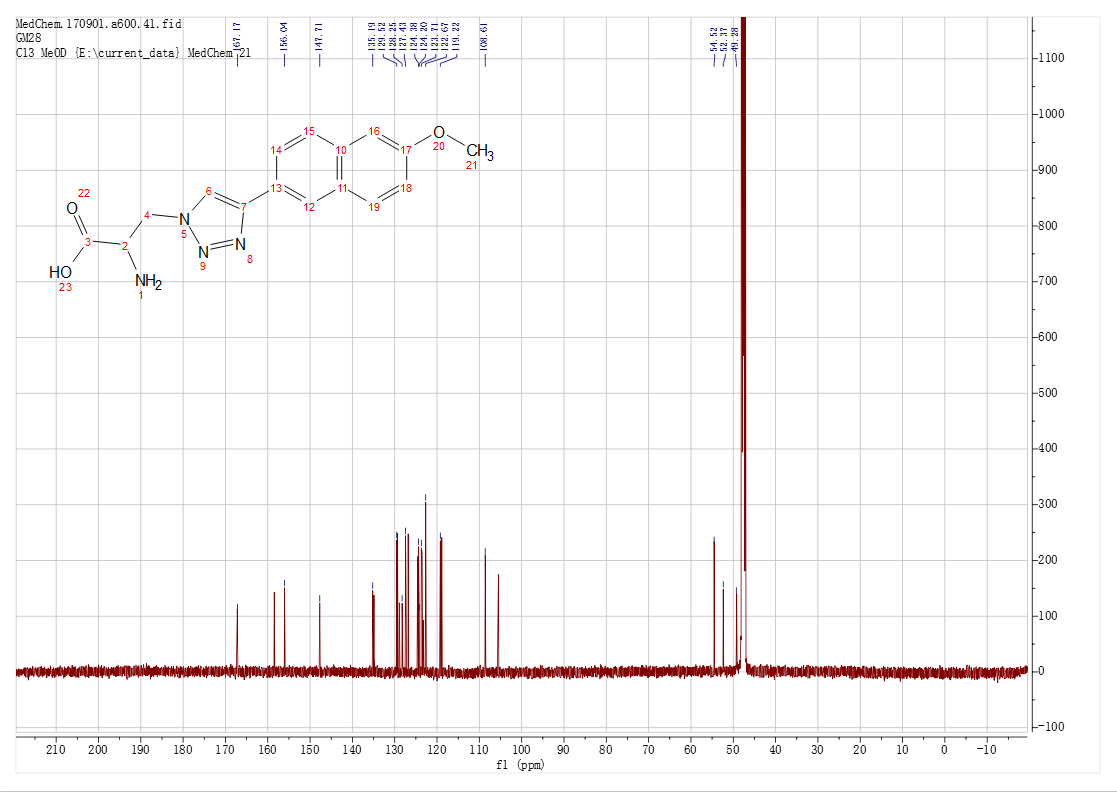


13f


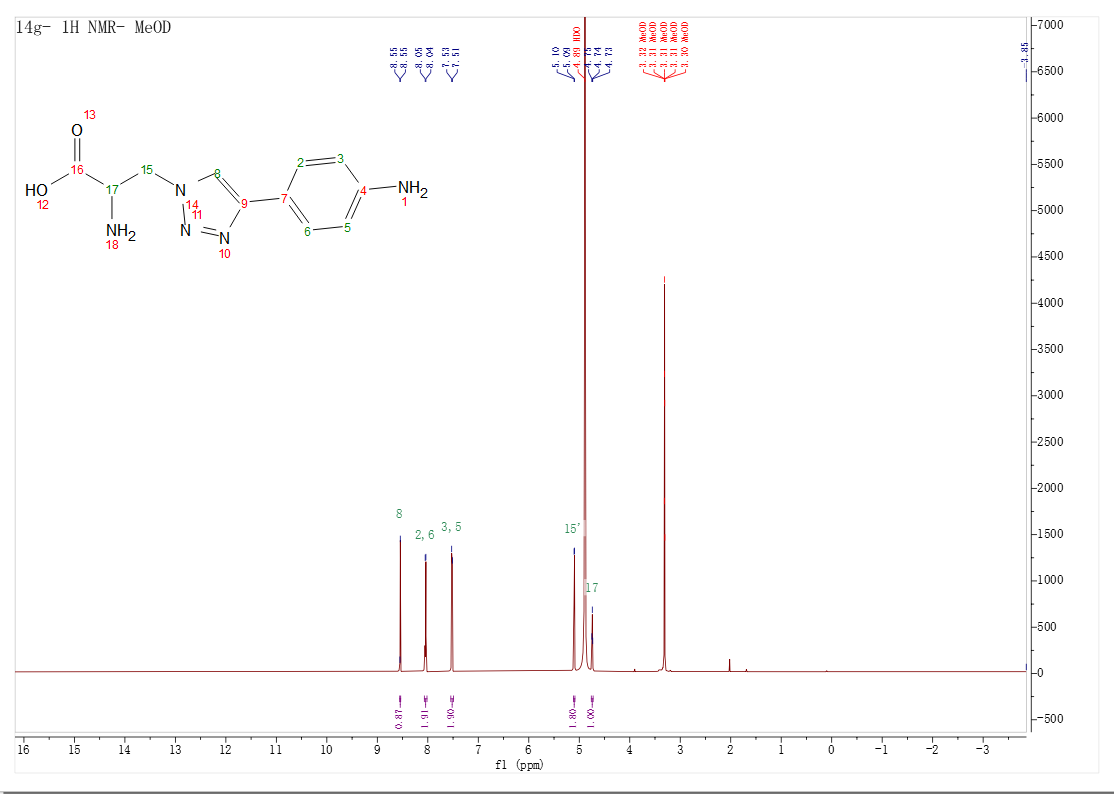


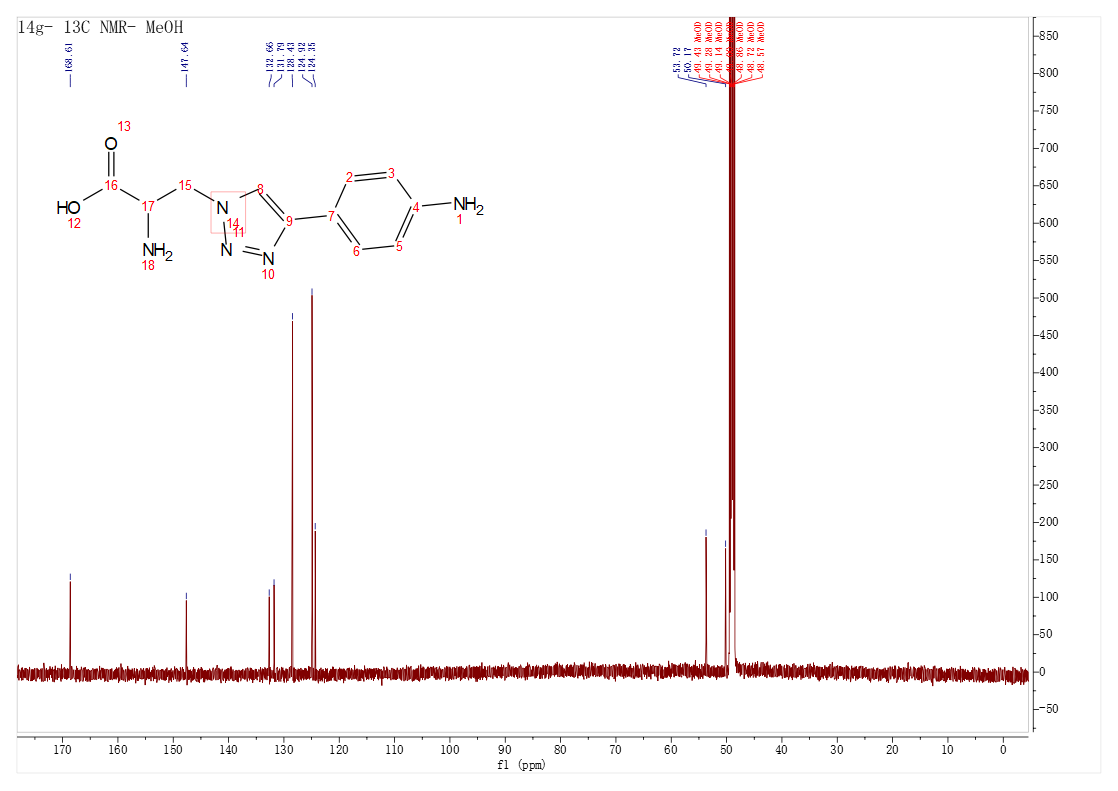


13g


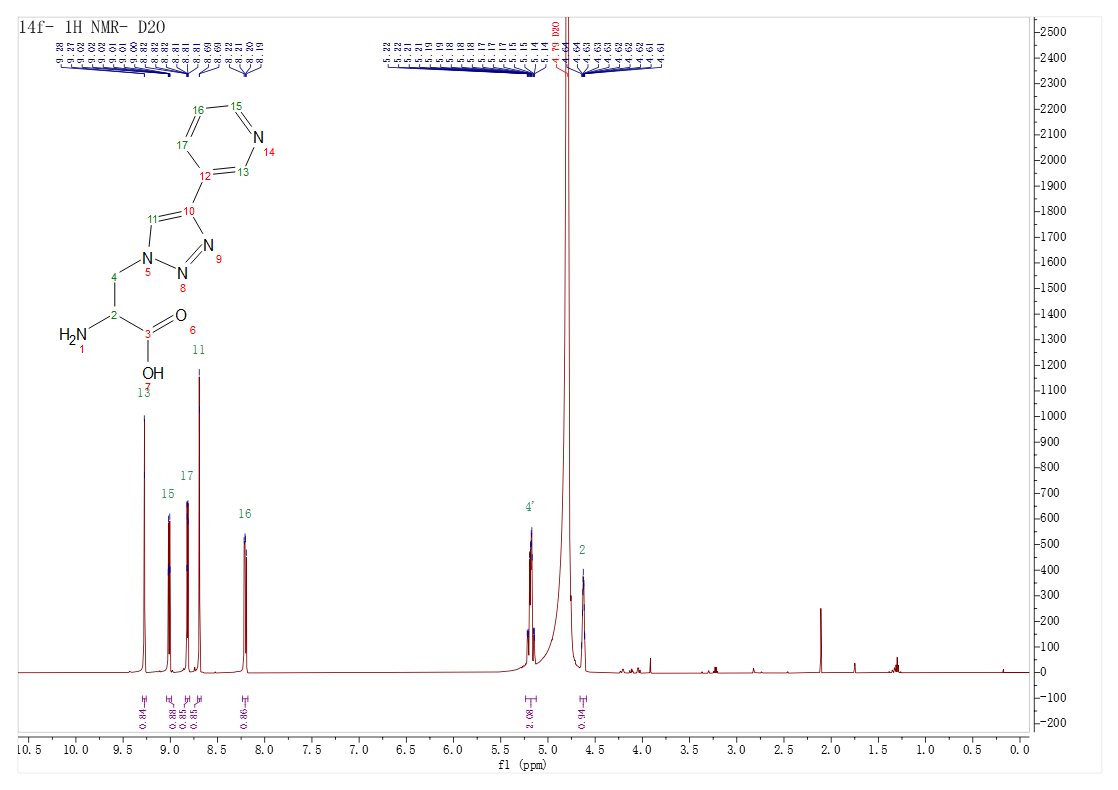


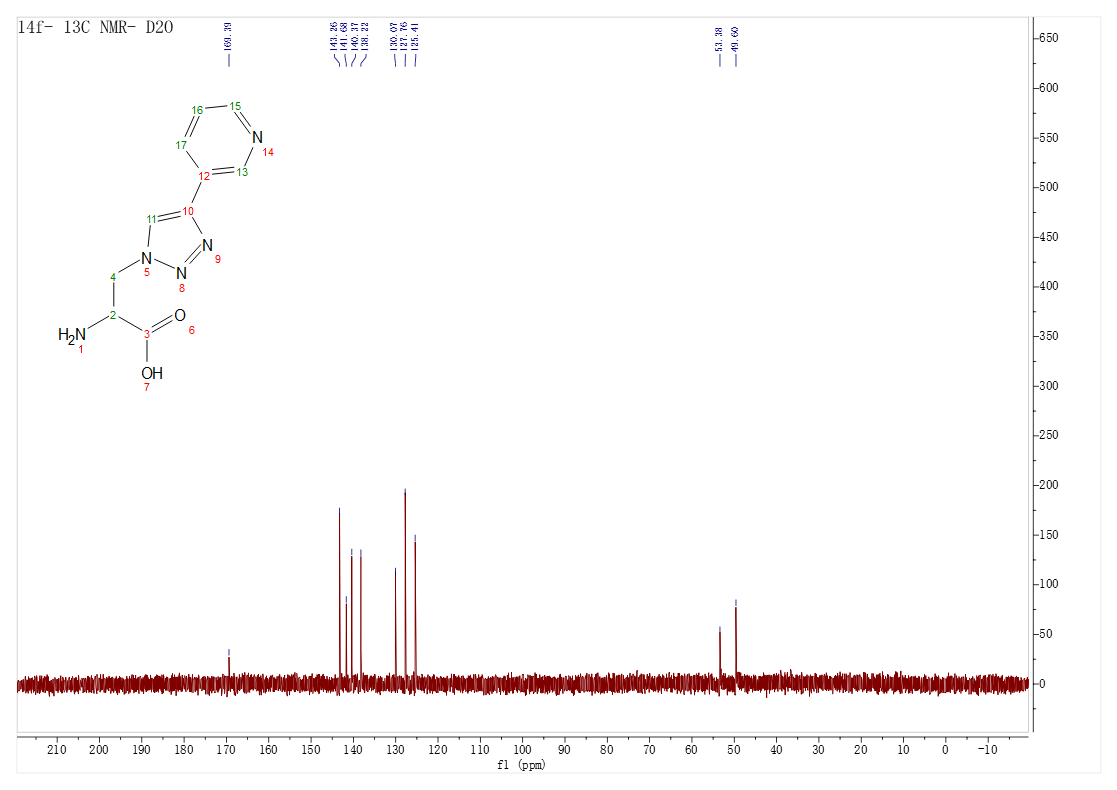


13h


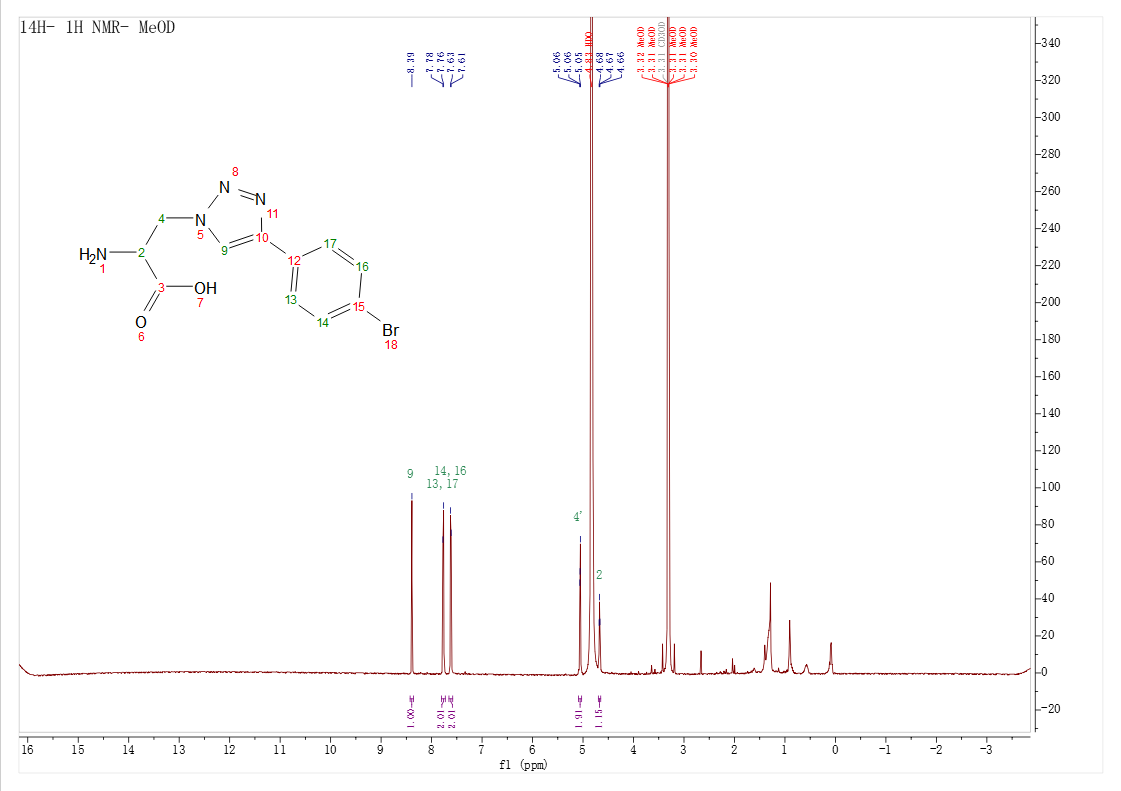


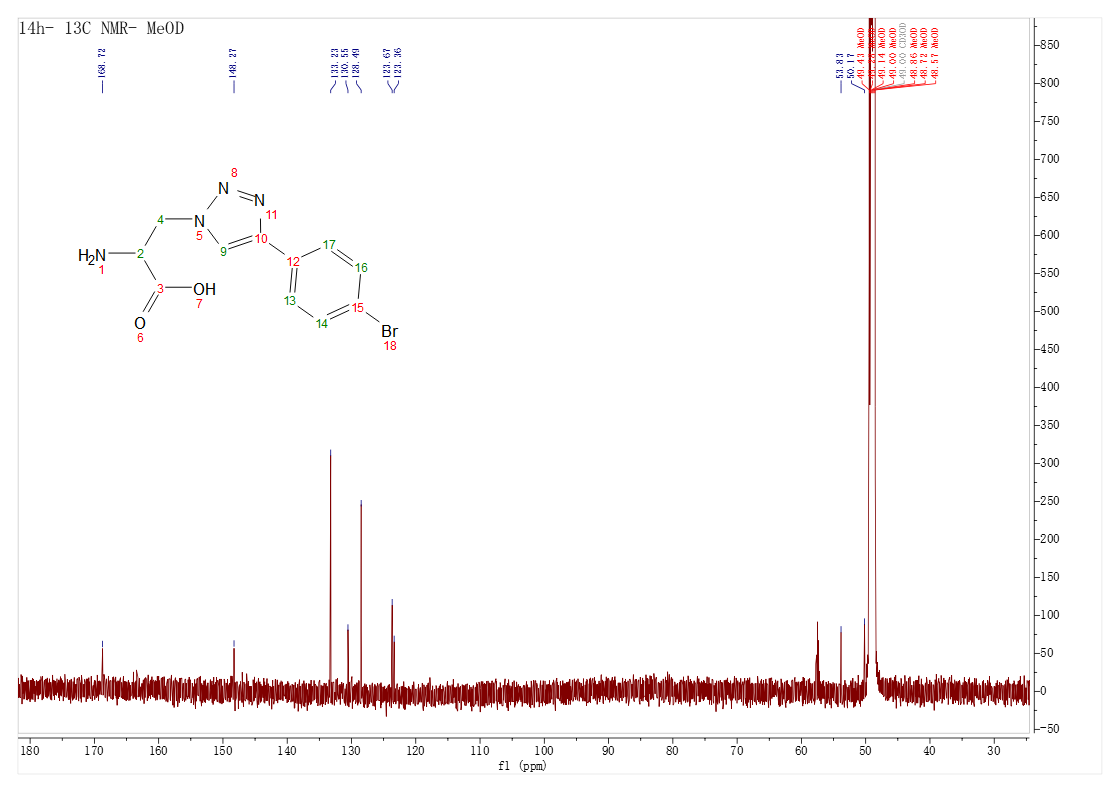


13i


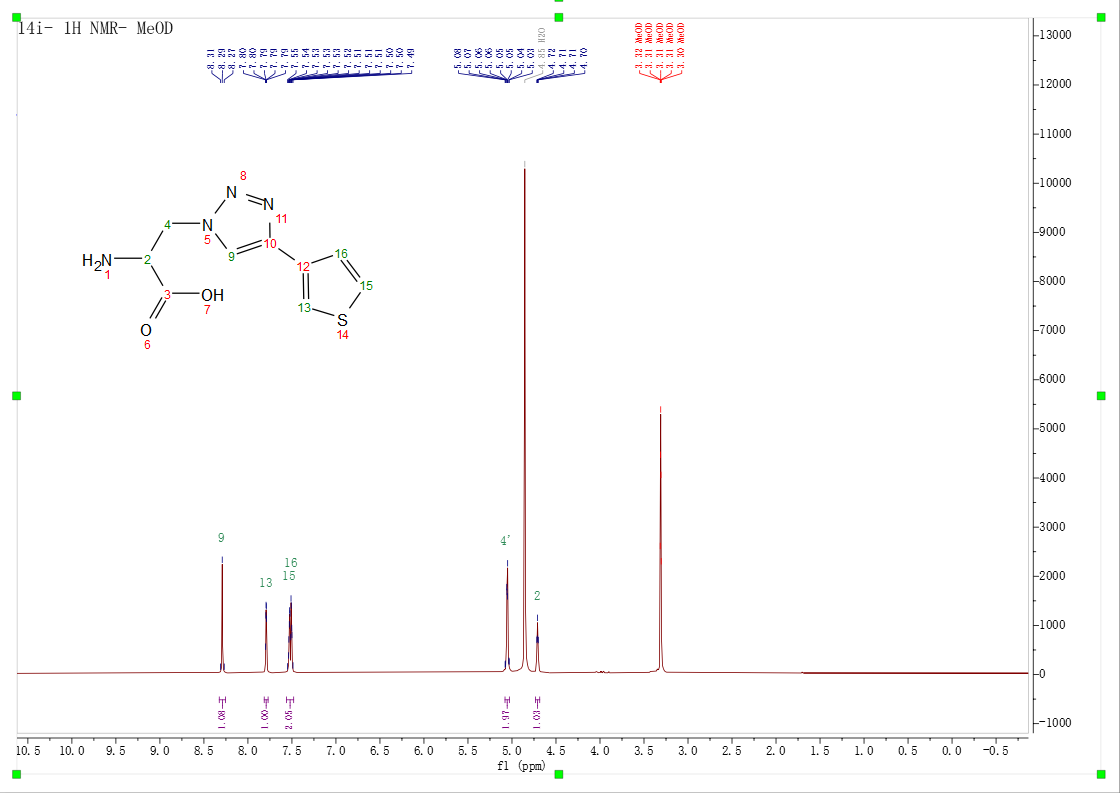


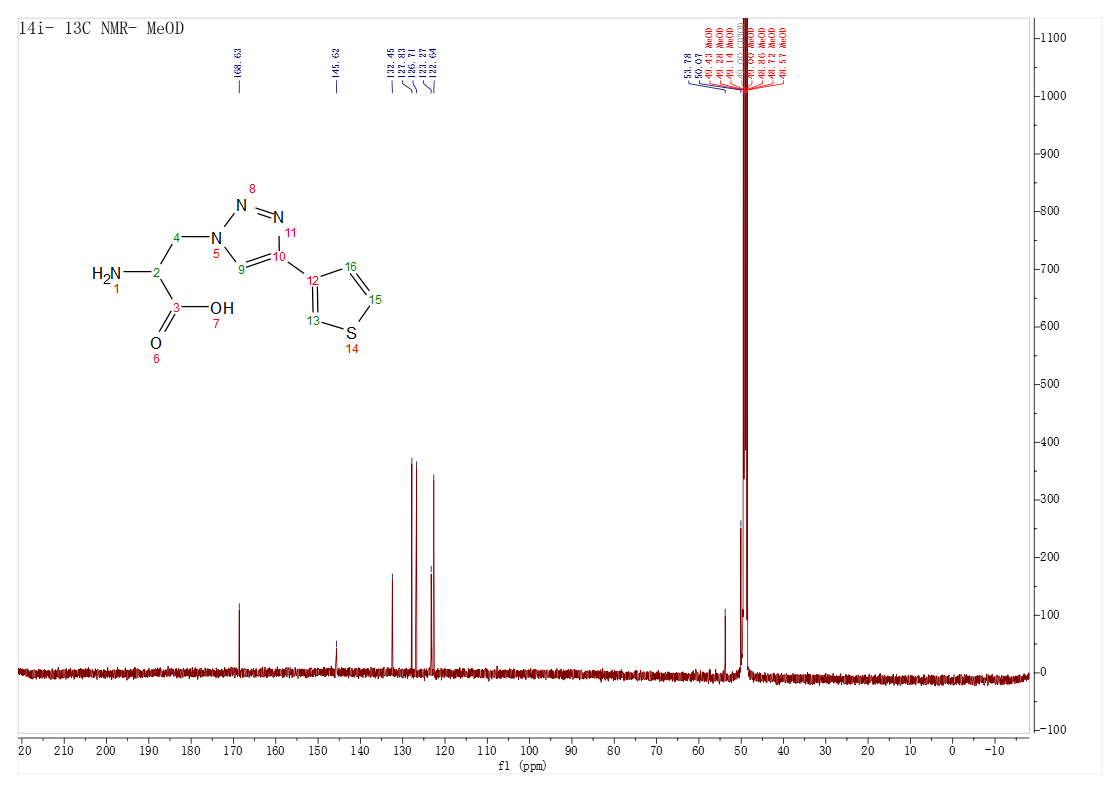


13j


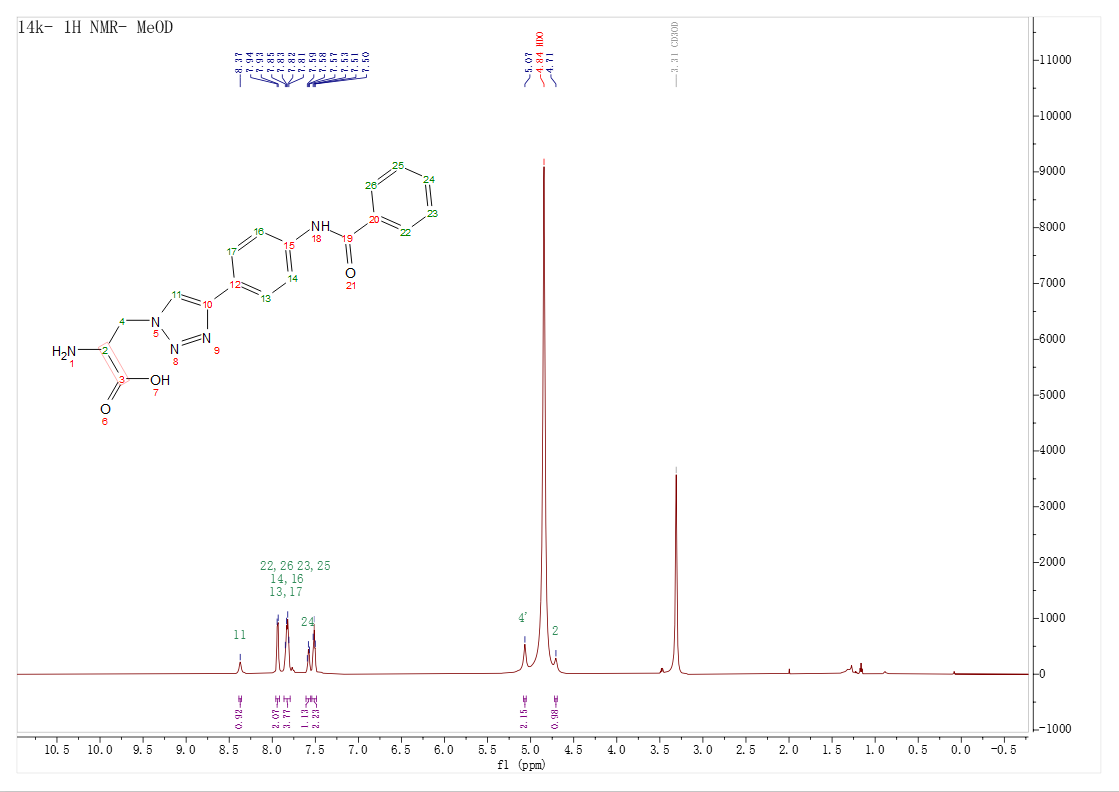


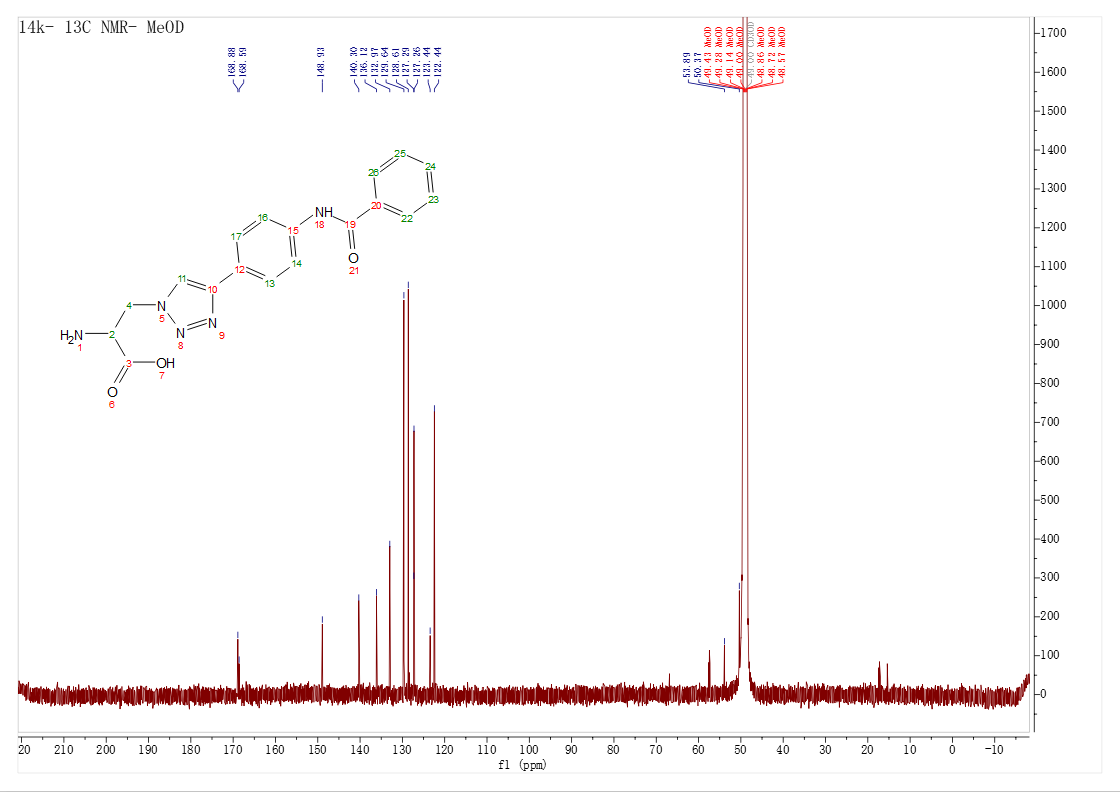


15


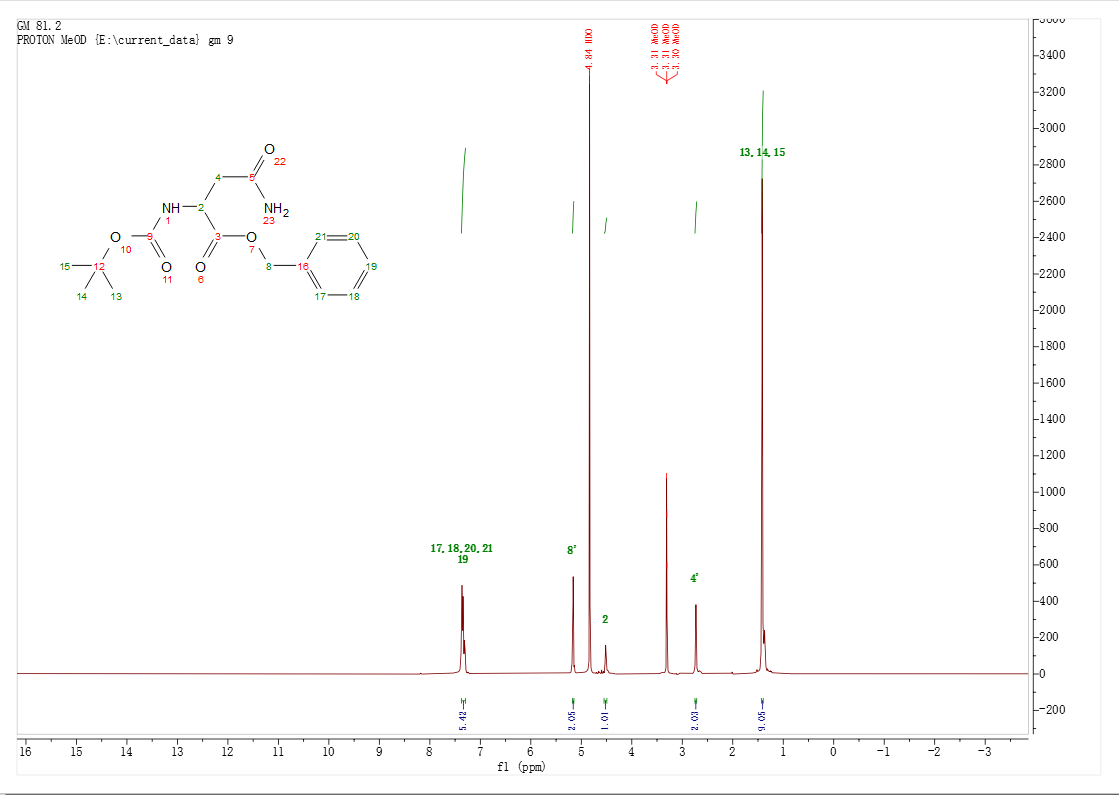


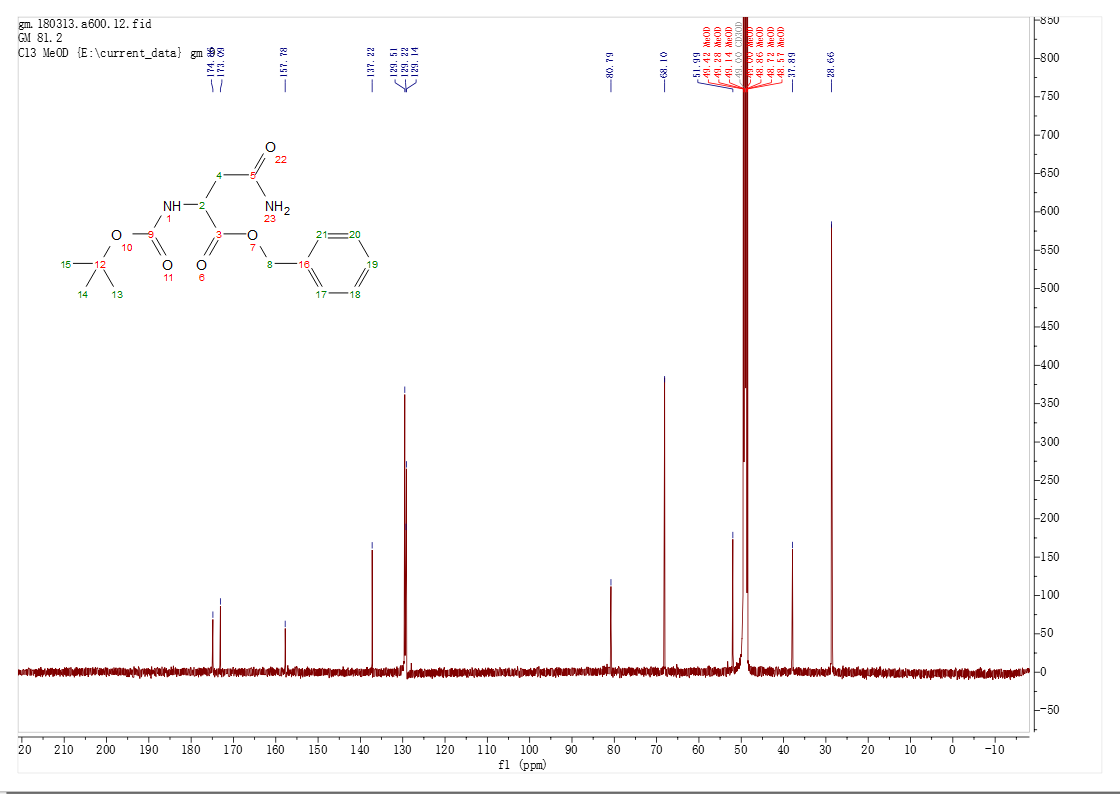


16


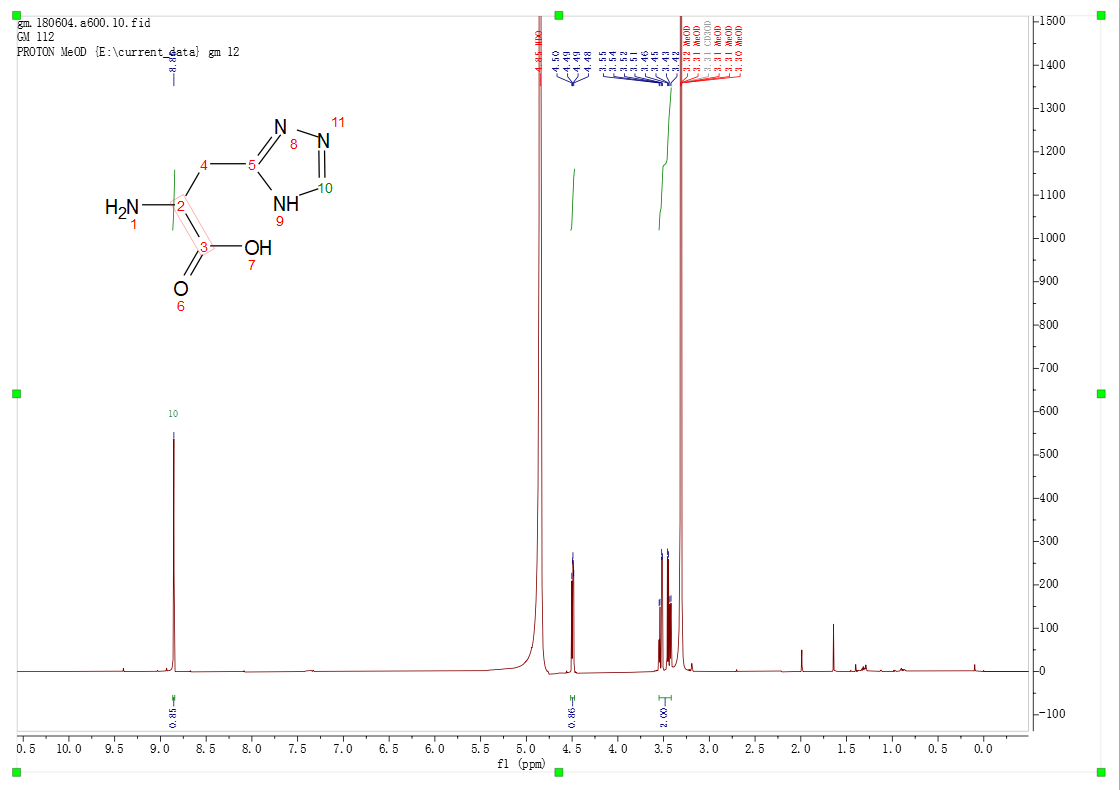


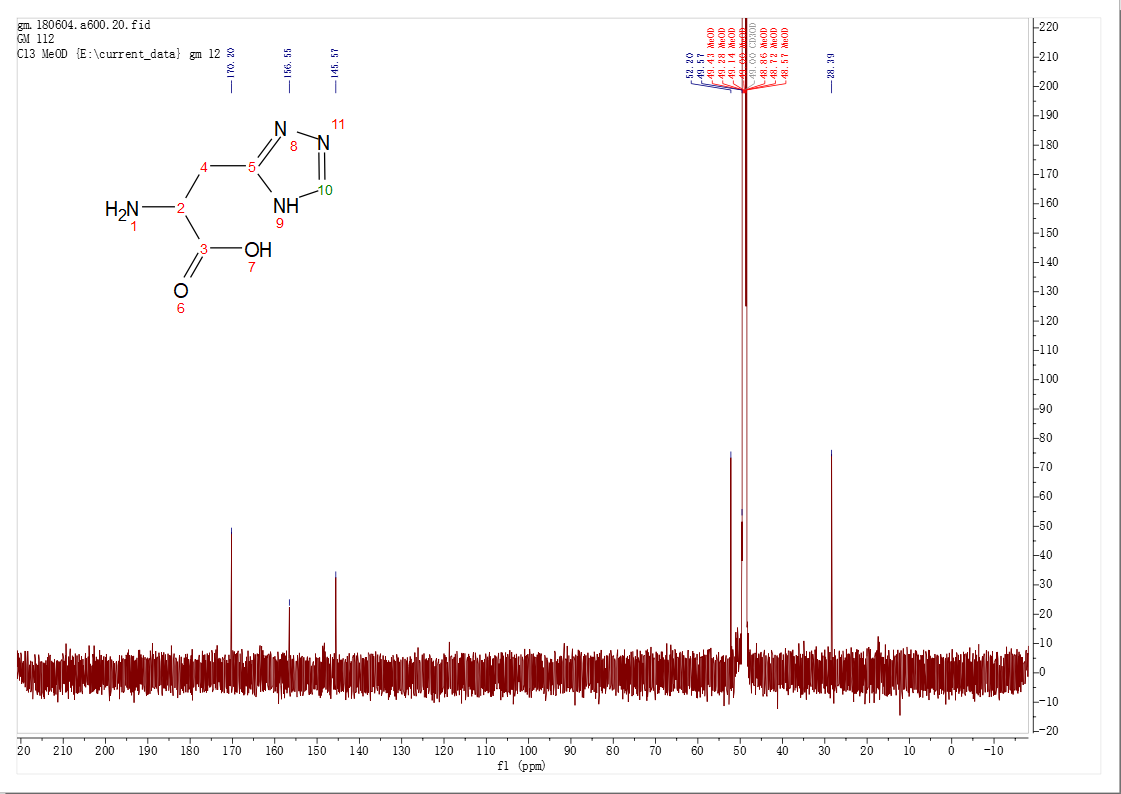

Supplement: Supplementary file 2 [file DataSheet1.DOCX]
